# Pro-Inflammatory Cytokines, IFNγ and TNFα, Influence Immune Properties of Human Bone Marrow and Wharton Jelly Mesenchymal Stem Cells Differentially

**DOI:** 10.1371/journal.pone.0009016

**Published:** 2010-02-02

**Authors:** S. Jyothi Prasanna, Divya Gopalakrishnan, Shilpa Rani Shankar, Anoop Babu Vasandan

**Affiliations:** Manipal Institute of Regenerative Medicine, Constituent Institute of Manipal University, Bangalore, Karnataka, India; Katholieke Universiteit Leuven, Belgium

## Abstract

**Background:**

Wharton's jelly derived stem cells (WJMSCs) are gaining attention as a possible clinical alternative to bone marrow derived mesenchymal stem cells (BMMSCs) owing to better accessibility, higher expansion potential and low immunogenicity. Usage of allogenic mesenchymal stem cells (MSC) could be permissible *in vivo* only if they retain their immune properties in an inflammatory setting. Thus the focus of this study is to understand and compare the immune properties of BMMSCs and WJMSCs primed with key pro-inflammatory cytokines, Interferon-γ (IFNγ) and Tumor Necrosis Factor-α (TNFα).

**Methodology/Principal Findings:**

Initially the effect of priming on MSC mediated suppression of alloantigen and mitogen induced lymphoproliferation was evaluated *in vitro*. Treatment with IFNγ or TNFα, did not ablate the immune-suppression caused by both the MSCs. Extent of immune-suppression was more with WJMSCs than BMMSCs in both the cases. Surprisingly, priming BMMSCs enhanced suppression of mitogen driven lymphoproliferation only; whereas IFNγ primed WJMSCs were better suppressors of MLRs. Further, kinetic analysis of cytokine profiles in co-cultures of primed/unprimed MSCs and Phytohematoagglutinin (PHA) activated lymphocytes was evaluated. Results indicated a decrease in levels of pro-inflammatory cytokines. Interestingly, a change in kinetics and thresholds of Interleukin-2 (IL-2) secretion was observed only with BMMSCs. Analysis of activation markers on PHA-stimulated lymphocytes indicated different expression patterns in co-cultures of primed/unprimed WJMSCs and BMMSCs. Strikingly, co-culture with WJMSCs resulted in an early activation of a negative co-stimulatory molecule, CTLA4, which was not evident with BMMSCs. A screen for immune suppressive factors in primed/unprimed WJMSCs and BMMSCs indicated inherent differences in IFNγ inducible Indoleamine 2, 3-dioxygenase (IDO) activity, Hepatocyte growth factor (HGF) and Prostaglandin E-2 (PGE2) levels which could possibly influence the mechanism of immune-modulation.

**Conclusion/Significance:**

This study demonstrates that inflammation affects the immune properties of MSCs distinctly. Importantly different tissue derived MSCs could utilize unique mechanisms of immune-modulation.

## Introduction

MSCs are plastic adherent cells expressing the minimal marker set of CD90, CD73 and CD105 and absence of CD14, CD79, CD34, CD45, and HLA-DR [1], [2]. MSCs home to injured sites and result in tissue regeneration in different injury models [3], [4]. These attributes of MSCs make them lucrative candidates for a cell based regenerative and repair therapy. Usage of MSCs as cell based therapeutics for repair is strengthened by the fact that MSCs appear to escape immune surveillance [5]. Human MSCs can be probably transplanted even against xenogenic barriers [6]. Bone marrow derived MSCs have been shown to modify or attenuate the effector functions of different immune populations like T cells, B cells, natural killer (NK) cells and dendritic cells (DC) subsets [7]–[13]. Owing to their property of immune-modulation, MSCs are being considered as double edged sword which can not only bring about tissue repair but also attenuate adverse inflammatory reactions in an allogenic setting. The immune-modulatory actions of MSCs have been tested *in vivo*. BMMSCs either reverse or prolong survival of mismatched grafts and decrease GVHD [14]–[18]. Despite these reports there are still contradictions in the literature on the immune status of MSCs. Studies by Elippoulous *et al.*, 2005 indicate that allogenic murine MSCs result in robust cell mediated immune responses in MHC class I and class II mismatched immuno-competent mice [19]. It is still not clear whether MSCs cause systemic immune suppression *in vivo* [20]. These discrepancies can be explained only when we understand the local environmental influences on immune behavior of MSCs.

MSCs have been identified and propagated from many different tissue sources [21]–[23]. Most of the tissue derived stem cells have common mesenchymal characteristics like cell surface marker expression, differentiation into mesenchymal lineages and immune suppressive behavior [24]–[27]. Despite commonalities amongst stem cells isolated from diverse sources, tissue specific influences on their stem cell behavior and immune properties cannot be negated. Of particular interest are fetal MSCs which are more primitive than the other tissue derived MSCs. MSCs derived from the Wharton's jelly or umbilical cord matrix is being considered as a potential alternative to bone marrow because of ease of isolation, higher expansion potential and greater accessibility of clinical samples than BMMSCs. Moreover bone marrow isolation is a painful and invasive procedure. Donor age differences have been shown to influence proliferative characteristics and differentiation capacity of BMMSCs [28]. Age related differences if any are minimal in WJMSCs samples as compared to other MSCs. WJMSCs are also immunosuppressive in *in vitro* lymphoproliferation experiments [29]. Human WJMSCs aided in neuronal regeneration in a rat model of spinal cord injury [30]. However, few reports suggest that not all fetal tissue derived MSCs have robust immune suppression properties as observed in adult stem cells [31], [32]. Thus it is important to have an in depth insight of the immunomodulatory behavior of MSCs derived from different sources differing in their primitiveness.

MSCs are stated to be hypo-immunogenic; however they do respond to inflammation. Inflammatory situation prevails during any tissue injury and MSCs would be exposed to such stimuli in many clinical conditions. The immune behavior of MSCs can be influenced not only by neighboring cells but also environmental factors like systemic or local inflammation as in case of Graft versus host disease (GVHD). Few recent reports in fact indicate the role of inflammatory cytokines in effecting immune functions of MSCs. IFNγ has been reported to enhance the immune-suppressive behavior of MSCs by upregulation of inhibitory molecule B7-H1 [33]. IFNγ induced IDO production by MSCs was shown to be the key player in immuno-suppression of T cells [34]. In comparison to wild type MSCs, murine MSCs from IFNγR1^−/−^ mice could not prevent GVHD [35]. In contrast, in a collagen induced mouse model of arthritis, MSCs did not cause beneficial effect in fact the report proved that TNFα secreted at the inflammatory site could cause reversal of immune-suppressive behavior [36]. IFNγ and TNFα are key pro-inflammatory cytokines mediating inflammatory events during several injury and pathological situations. MSCs would be exposed to these stimuli in an *in vivo* inflammation/transplantation scenario. We have thus attempted to re-evaluate the immune properties of MSCs from bone marrow and Wharton's jelly when primed with inflammatory stimuli, IFNγ and TNFα The results discussed in the paper highlight that although most MSCs from different tissue sources are reported to be immune suppressive, they adopt unique mechanisms for immune-modulation possibly due to inherent differences in immune-suppressive factors expressed. Further the study proposes that different inflammatory factors modulate the immune-regulatory capacity of MSCs distinctly.

## Materials and Methods

### Antibodies

Anti human -MsCD73 PE, -MsCD90 PE, -MsCD105 PE, -MsCD166 PE, -MsCD34 PE, -MsCD45 FITC, -MsCD19 PE, -MsHLA-DR FITC, -MsHLA-ABC FITC, -MsCTLA4-FITC, -MsCD50 FITC, -MsCD54 PE, -MsCD80 FITC, -MsCD86 PE, -MsCD95 FITC, the relevant isotype controls, purified anti-human -MsCD28, -MsCD69, -MsCD45RA, and -MsCD45RO were obtained from BD Pharmingen, San Jose, CA,USA. Anti human -goat FABP4, anti human -Ms Osteocalcin and -Ms Collagen II were obtained from R&D SYSTEMS, Inc. Purified anti human -MsSSEA4, rabbit anti-mouse IgG_1_ FITC and rabbit anti-goat IgG_1_ FITC secondary antibodies used to detect the primary antibodies were from Chemicon, Temecula, CA, USA.

### Culture of Human Bone Marrow Derived MSCs

Bone marrow aspirates were drawn from iliac crest of donors from Manipal hospital, Bangalore, India. Informed written consent was obtained using an Institutional Review Board approved protocol (Protocol No: MHB/SCR/010) from Ethics committee of Manipal Hospital and Manipal Heart Foundation, Bangalore, India. Bone marrow samples were diluted and layered on Lymphoprep™ (1.077 g/ml) obtained from Axis-shield PoC AS (Norway) following which the sample was spun at 400 g for 20 minutes at 22°C. The buffy coat containing the mononuclear cells was washed with Dulbecco's phosphate buffered saline (DPBS) from GIBCO-BRL, USA and plated in KO-DMEM (GIBCO-BRL,USA) containing 10% FBS (HyClone, Utah, USA), 2 mM L-Glutamine (GIBCO-BRL,USA) and antibiotic-anti-mycotic (GIBCO-BRL,USA). The adherent cells were propagated in the above mentioned media in a humidified incubator maintained at 37°C with 5% CO_2_. The initial plating densities and seeding densities for propagation was as previously described [37], [38]. Bone marrow derived cells were characterized for the presence of mesenchymal markers (CD73, CD44, CD90, CD105) and absence of CD34 and CD45 before performing different experiments (Figure S1A). All experiments were set up within passage 10 of MSC culture.

### Culture of Human Wharton's Jelly Derived Mesenchymal Stem Cells

Human umbilical cords were obtained from full term women immediately after delivery from gynecology department of Manipal hospital, Bangalore, India. Informed written consent was obtained after getting an approval (Protocol No: MIRM/001/08) from Institutional Ethics committee of Manipal Hospital and Manipal Heart Foundation, Bangalore, India. After thorough washing with DPBS containing antibiotics the cord was cut open and the arteries and veins were removed. The tissue was treated with Collagenease blend (Sigma, St.Louie, USA) at a net concentration of 500 µg/ml for 20 hr. Post-digestion with collagenease the cells were filtered through a 100 µ cell strainer (BD FALCON, Bedford, USA) to remove tissue debris. Cells were then washed with DPBS and cultured in KO-DMEM containing 10% FBS, 2 mM L-Glutamine and antibiotics. WJSCs were grown till confluence and cells between Passage 3- Passage 8 were used for setting experiments after characterizing for mesenchymal stem cell markers (Figure S1A).

### Peripheral Blood Mononuclear Cells (PBMC) Culture and Stimulation

Blood for PBMC culture and stimulation was obtained from healthy donors after taking written consent using a protocol (Protocol no. MIRM/ASC-2/08) approved by Institutional Ethics committee of Manipal Institute of Regenerative Medicine, Manipal University, Bangalore. PBMCs were separated from heparinized blood of donors using Lymphoprep™. PBMCs (1×10^6^ cells/ml) were cultured in 96 well plates (BD FALCON, NJ,USA) in RPMI-1640 (GIBCO-BLR,USA)supplemented with 2 mM glutamine, 10% FBS and antibiotic-anti-mycotic. For mitogenic stimulation experiments, PBMCs were treated with Phytohaemagglutinin (PHA) (Biological Industries, Israel) at a concentration of 20 µg/ml for different time intervals (12 to 96 hr).

Mixed lymphocyte reactions were set up in 96 well BD FALCON plates by co-culturing the responder PBMCs (1×10^6^ cells/ml) with stimulator PBMCs (0.5×10^6^ cells/ml) from different donors or an autologous control for 6 days in RPMI-1640 medium with 10% FBS and antibiotics as described above. The stimulator PBMCs were treated with 10 µg/ml mitomycin C (Sigma, St.Louie, USA) for 2 hr and were thoroughly washed with DPBS before using them for Mixed Lymphocyte Reaction (MLR) assay. Cell Proliferation resulting from both mitogenic and allogenic stimulation of PBMCs was measured by a BrdU cell proliferation assay kit (Calbiochem, USA).

### MSC Priming

Bone marrow derived MSCs and Wharton's jelly derived MSCs were primed with inflammatory cytokines 150 U/ml of IFNγ (Sigma, St.Louie, USA) and 10 ng/ml of TNFα (Sigma, St.Louie, USA) for 72 hrs and subsequently analyzed for cell surface markers. The dosage for IFNγ and TNFα treatment of MSCs was decided based on published reports [39], [40], [41]. Optimal time of induction was chosen based on kinetic analysis of HLA-ABC and HLA-DR surface expression (Figure S2). Primed and unprimed MSCs were thoroughly washed after stimulation, either mitomycin C treated (10 µg/ml) or untreated, and used for co-culture with PBMCs in lymphoproliferation experiments. The dosage of Mitomycin C used was affective in causing growth arrest of MSCs (Figure S3).

### Tri-Lineage Differentiation

BMMSC and WJMSC cells primed/unprimed with IFNγ or TNFα (72 hr) were washed with DPBS and cultured in adipogenic, osteogenic or chondrogenic inductive media (media change was done every 3 days) to evaluate their mesenchymal differentiation potential.

Adipogenic inductive media contained 1.0 µM dexamethasone (Sigma Aldrich), 0.5 mM isobutylmethylxanthine (Sigma Aldrich), 1.0 µg/ml insulin (Sigma Aldrich) and 100 µM indomethacin (Sigma Aldrich) in DMEM-KO supplemented with 10% FBS. Oil deposition in differentiated adipocytes was evaluated by Oil O Red staining after 18 days.

Osteogenic inductive media contained 10^−8^ dexamethasone (Sigma Aldrich), 50 µg/ml ascorbic acid (Sigma Aldrich), 10 mM β-glycerophosphate (Sigma Aldrich) in DMEM KO supplemented with 10% FBS. Mineralization was detected by Von Kossa staining after 18 days.

Chondrogenic differentiation was induced in confluent monolayer cultures using chondrogenic differentiation media from Lonza, Basal, Switzerland. Proteoglycan deposition was checked by Safranin O staining after 15 days of induction [42].

**Figure 1:**
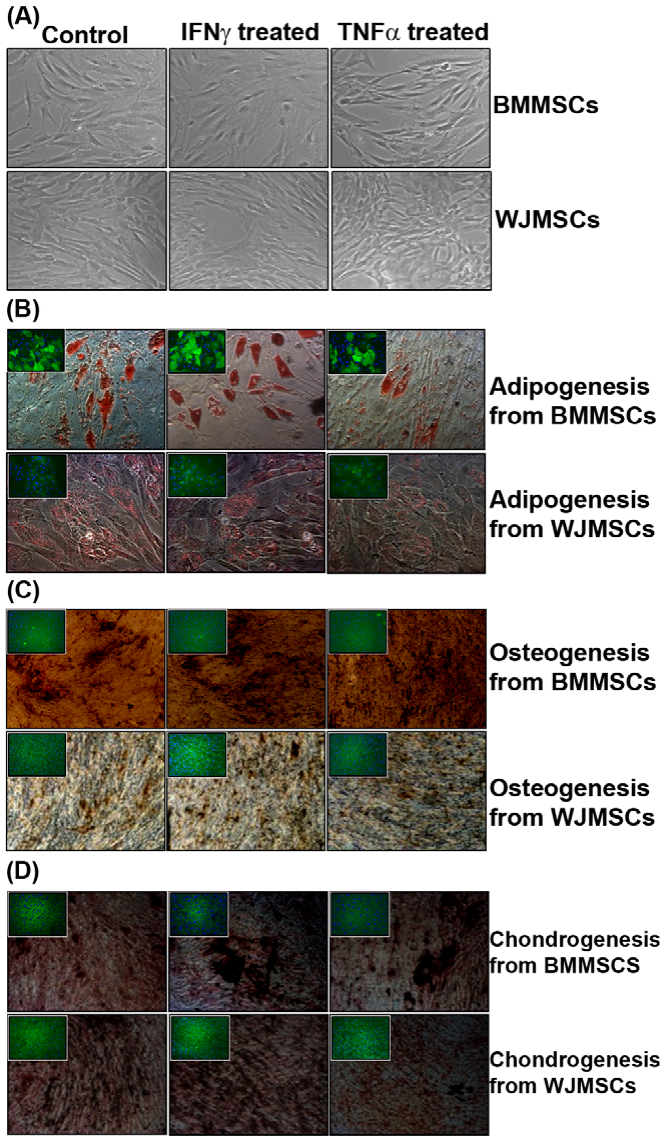
Phenotypic changes and tri-lineage differentiation potential of human MSCs on exposure to IFNγ and TNFα. (A) Phase contrast pictures of MSCs, captured by a Nikon Eclipse TE2000S microscope (magnification 10×10), depicting phenotypic changes on exposure to pro-inflammatory cytokines. (B) Lipid droplets stained with Oil O Red depicting adipogenesis {Inset: FABP4 staining of MSC- derived adipocytes} (magnification 20×10) (C) Osteogenisis depicted by Von Kossa staining of calcium mineralized deposits {Inset: Osteocalcin staining of MSC derived mature osteocytes} (magnification 10×10). (D). Sulfated proteoglycans stained with Safranin O demonstrating chondrogenesis {Inset: Collagen II staining of MSC derived chondrocytes} (magnification 10×10). Control refers to cells unexposed to pro-inflammatory cytokines.

Differentiation pictures depicted in Figure 1 are from MSCs at Passage 5.

Additionally, Immuno-fluorescence staining was performed with FABP-4, Osteocalcin and Collagen II specific antibodies to ascertain adipogenic, osteogenic and chondrogenic differentiation of MSCs respectively, post induction with differentiation media.

### Immuno-Cytochemistry

MSCs were cultured and primed with IFNγ/TNFα in BD FALCON chamber slides. Post priming MSCs were induced with specific differentiation media for either 15 or 18 days according to protocols mentioned above. Subsequently, cells were fixed with 4% para-formaldehyde (Sigma) containing 5% Sucrose for 20 minutes at room temperature and washed twice with DPBS. Cells were permeablized and blocked with 0.1% Triton X-100 containing 3% BSA in DPBS for 30 min. Staining with specific primary antibodies was performed in DPBS containing 0.1% BSA overnight at 4 degrees. Primary antibody staining was detected using appropriate FITC conjugated secondary antibodies diluted in DPBS containing 0.01% BSA. Cells were incubated in secondary antibody for 45 minutes at room temperature. Cells were finally washed thrice with 0.01% Tween 20 containing 0.1% BSA in DPBS and counterstained with 4', 6'-diamidino-2-phenylindole dihydrochloride (DAPI) for 5 minutes. Fluorescent images were captured using a Nikon Eclipse 80i (Nikon) microscope and analyzed using Q imaging software.

### Population Doublings

Population doublings for each passage was calculated after the cells reached 90% confluence using the formula lgNt–lgN_0_/lg2 [43]. Nt is the harvested cell number and N_0_ is the initial no. of cells seeded at each passage. The mean cell no. was calculated by trypan blue exclusion method (Figure S4).

### Cytogenetics

The cytogenetic stability of BMMSCs and WJMSCs was evaluated at passage 5 of culture with and without priming with inflammatory cytokines by an independent diagnostic centre (Anand diagnostics, Bangalore, India). 20 metaphases were screened and analyzed by GTG banding.

### MSC-PBMC Co-Cultures

Human bone marrow derived MSCs and Wharton's jelly derived MSCs, mitomycin C treated, were co-cultured with donor PBMCs at ratios of 1∶10 and 1∶100 (MSC: PBMC ratio) (referred to as 1% and 10% MSCs respectively in the tables and figures). Proliferation rates were measured after 80 hr by BrdU cell assay kit (Calbiochem) as per the kit protocol. Similar co-cultures were set up even with MSCs primed by IFNγ and TNFα. MSCs alone and PBMCs alone were used as controls.

To check whether MSCs (primed/unprimed) influenced the PBMC proliferation status, allogenic stimulation or mitogenic stimulation of PBMCs was performed in presence of MSCs. MSCs were allowed to adhere in the 96 well plates for 30 minutes before addition of PBMCs along with PHA (mitogenic stimulation) or mitomycin C treated stimulator PBMCs (in case of MLRs). The proliferation was checked using BrdU incorporation as mentioned above. Proliferation was measured after 80 hr in case of mitogenic stimulation experiments and for 6 days in case of MLRs. The fold suppression depicted in the tables was relative to PHA treated PBMCs or PBMCs treated with stimulator PBMCS which was considered as 100.

Culture supernatants were collected after different time intervals in co-culture experiments set up in 96 well culture plates and PBMCs were collected at the same time points mechanically for cell surface analysis using flow cytometry. MSCs at passage 6 were used for this set of experiments.

### Cytokine ELISAs

Supernatants from mitogen stimulated cultures were collected after different time intervals and stored at −20 degrees and the samples were subsequently assayed in triplicates for specific cytokines using respective cytokine kits (OptEIA Human ELISA; BD Biosciences, CA, USA) according to manufacturer's instructions. Optical Density (OD) was measured at 450 nm in the Multilabel counter (VICTOR3™ Multilabel Counter model 1420–032, Perkin Elmer).

For analysis of HGF and PGE2 levels supernatants were obtained from control and primed MSC cultures after 72 hr. Secreted levels of HGF and PGE2 was quantified using cytokine ELISA kits from R&D Systems according to the kit protocols.

### Real Time RT-PCR Analysis

Total RNA from BMMSCs and WJMSCs was isolated using TRIzol (Invitrogen) and cDNA was synthesized using SuperScript III First- Strand kit (Invitrogen). Real time RT-PCR analysis was performed using Platinum SYBR Green qPCR SuperMix-UDG kit (Invitrogen). All the above methods were executed according to the manufacturer's instructions. Real time analysis of *IDO*, *HGF*, Cyclooxygenase-2 (*COX2*), Class II trans-activator (*CIITA*) and Glyceraldehyde-3-phosphate dehydrogenase (*GAPDH*) was done in duplicates on an ABI 7500 Real-time PCR system (Applied Biosystems). Reactions were set at 50°C for 2 minutes, 95°C for 10 minutes followed by 40 cycles of 95°C for 15 sec and the specific annealing temperatures (refer Table S1) for 1 minute. A dissociation step was incorporated for all analysis. SDS v1.4 software was used to analyze the results. Average C_T_ was calculated for different genes. ΔC_T_ values depicted in the table were calculated by subtracting the C_T_ of test from C_T_ of endogenous control (*GAPDH*). Cycle threshold above 35.0 were not considered for the analysis.

### IDO Activity Assay

IDO catalyses the conversion of Tryptophan to N-Formyl Kynurenine, which is catabolised to Kynurenine. Kynurenine levels in the supernatants are directly proportional to IDO activity. Culture supernatants from BMMSC and WJMSCs untreated or treated with IFNγ or TNFα were freshly harvested after 72 hr of priming and conversion to Kynurenine was evaluated. 100 µl of culture supernatants were treated with 50 µl of 30% trichloroacetic acid (Sigma Aldrich), vortexed and the tubes were centrifuged at 930 g for 5 min, the supernatant obtained were transferred to 96well plate followed by addition of equal volume of Ehrlich reagent (Sigma Aldrich) (1% p-dimethylbenzaldehyde in glacial acetic acid). OD was measured at 490 nm in Multilabel counter (VICTOR^3^™ Multilabel Counter model 1420–032, Perkin Elmer). The amount of Kynurenine was determined using a standard curve of Kynurenine (0–2500 µM) [44].

### Flow Cytometry

**Figure 2:**
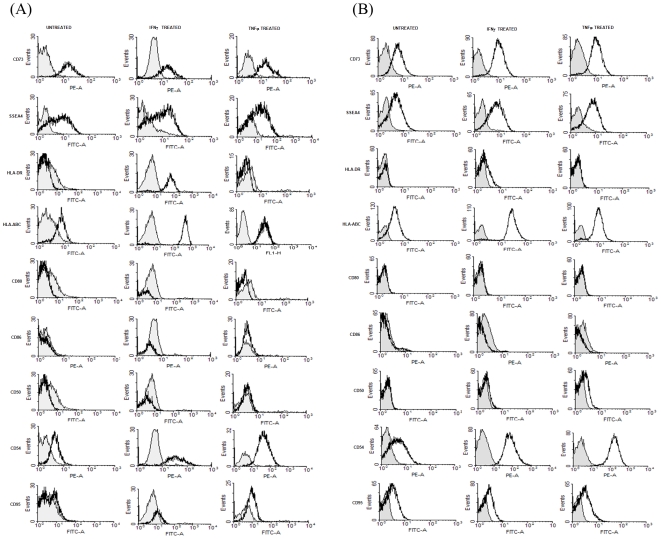
Inflammatory cytokines affect the immune-phenotype of human MSCs derived from bone marrow and Wharton's jelly. BMMSC's (A) and WJMSC's (B) cultured with or without IFNγ or TNFα for 72 hr were stained with appropriate conjugated antibodies and the surface expression was monitored by flow cytometry. Grey filled histograms represent specific isotype controls. Data is representative of three experiments performed with three independent donor MSCs.

Cell surface markers on MSCs were analyzed using a panel of antibodies as mentioned above. Non-specific florescence was determined with appropriate isotype controls. PHA activated PBMCs alone (indicated as ‘P+P’ in the figures) and PHA activated PBMCs co-cultured with primed/unprimed MSCs (indicated by ‘+’ and the co-culture condition in the figures) were washed with PBS and stained in antibody containing buffer (DPBS containing 1% FBS and 0.01% sodium azide) on ice for 45 minutes. For indirect staining cells were washed and treated with appropriate secondary antibody for 30 minutes on ice. Stained cells were subsequently washed and fixed with 1% PFA and acquired on a BD LSR II flow cytometer and the data was analyzed using the WinMDI software (Figure 2).

### Statistical Analysis

Results are represented as means ± Standard deviation. Student's t-test was used to make statistical comparisons. *p-*value of less than 0.05 (*) and less than 0.01(**) were considered to be statistically significant.

## Results

### Stromal Cells Isolated from Human Bone Marrow and Wharton's Jelly Exhibit Conserved Mesenchymal Characteristics

Although isolation procedures for BMMSC and WJMSCs differ the conventional Mesenchymal characteristics, reported, like cell surface marker expression or the mesenchymal lineage differentiation capacity remains conserved. Both MSCs were positive for CD73, CD90, CD105, CD166 and negative for CD34, CD45, CD19 and HLA-DR (Figure 2, Figure S1). Interestingly smaller oil droplets were observed in WJMSC derived adipocytes as compared to adipocytes derived from BMMSCs (Figure 1, Figure S1B).

### MSCs from Bone Marrow and Wharton's Jelly Respond to Inflammatory Stimuli

Changes in morphology of BMMSCs were observed on addition of pro-inflammatory cytokines, IFNγ and TNFα. IFNγ primed BMMSCs looked bigger in size, had high amount of cytoplasm and a flattened irregular shape. Whereas, TNFα primed cells appeared more spindle shaped as compared to the control untreated BMMSCs. However, WJMSCs did not show marked morphological changes on treatment with pro-inflammatory cytokines consistently (Figure 1A). IFNγ is known to cause growth suppression in several cell types (Chan KW et al., 2008). Thus, the proliferation potential was checked in primed and unprimed MSCs at different passages. An increase in population doublings of TNFα primed BMMSCs was observed whereas IFNγ priming decreased the proliferative potential of BMMSCs only (Figure S4).

Despite changes in morphology the differentiation capacity of BMMSCs and WJMSCs to mesenchymal lineages was not compromised upon exposure to IFNγ and TNFα. (Figure 1B, C, D). No chromosomal abnormalities were observed in primed and unprimed cells (Figure S5). MSCs primed with inflammatory cytokines, IFNγ and TNFα, exhibited a normal karyotype suggesting cytogenetic stability of the cells.

MSCs from bone marrow and Wharton's jelly were then compared for different immunologically relevant markers upon treatment with IFNγ and TNFα Both the MSCs studied expressed stem cell markers CD73 and SSEA4 and their expression was not altered on treatment with IFNγ or TNFα. BMMSCs and WJMSCs express low levels of MHC class I but lack expression of HLA-DR and co-stimulatory ligands, CD80 and CD86. Expression of other crucial immune-stimulatory ligands; CD54 and CD50, and FAS (CD95) were also analyzed. BMMSCs and WJMSCs express significant levels of immune-adhesive ligand CD54 on their surface which was induced greatly by IFNγ and TNFα (Figure 2A and B). However, CD95 and CD50 were not detected on MSCs even upon treatment with inflammatory stimuli. The expression of stem cell markers, CD73 and SSEA4 does not change significantly on treatment with either IFNγ or TNFα with both the MSCs tested. HLA-ABC was induced majorly (Figure 2A, B) on treatment with pro-inflammatory cytokines. However, HLA-DR expression was upregulated on BMMSCs only on treatment with IFNγ but not with TNFα. HLA-DR was not detected on WJMSCs upon treatment with either IFNγ or TNFα.

This suggests that although MSCs from different tissue sources have more or less conserved immune properties minor differences exist in how they respond to inflammation.

### Immunogenicity of BMMSCs and WJMSCs Primed with Inflammatory Stimuli

MSCs have been reported to exhibit low immunogenicity when co-cultured with allogenic PBMCs [45], [46]. However, the immunogenicity of IFNγ and specially TNFα treated MSCs has not been extensively tested. The ability of allogenic WJMSCs and BMMSCs to elicit PBMC proliferation was tested in one-way MLRs with mitomycin C treated MSCs as stimulators. MSCs were either primed or unprimed with IFNγ or TNFα for the MLR reaction. As indicated in Table 1 mild proliferation was observed with BMMSCs treated or untreated with IFNγ. WJMSCs did not cause proliferation of allogenic PBMCs over and above the autologous control PBMCs, except in case of TNFα primed WJMSCs were mild proliferation was observed even at 1% cell dosage (*p*-value <0.05). Immunogenicity of MSCs seems to depend on the dosage of MSCs which interact with PBMCS. Similar results were obtained with different isolates of BMMSCs and WJMSCs. Thus it appears that inflammation does not majorly affect the allo-recognition of MSCs.

**Table 1:** BMMSCs and WJMSCs primed with inflammatory stimuli mildly effect proliferation of allogenic PBMCs.

Conditions	% proliferation ± S.D	
	BMMSC (P5)	WJMSC (P5)
Allogenic PBMCs	100.0	100.0
PBMCs alone	7.4±0.01	7.4±0.01
+10% Control MSCs	14.8±3.6	6.6±4.7
+1% Control MSCs	4.6±6.8	*0.1±0.01*
+10% IFNγ treated MSCs	14.9±4.1	4.4±2.8
+1% IFNγ treated MSCs	5.1±2.2	1.0±0.08
+10% TNFα treated MSCs	9.7±2.3	16.5±6.6
+1% TNFα treated MSCs	3.0±0.8	*12.1±4.3*

“+” refers to co-culture of allogenic PBMCs with either 1% or 10% dosage of primed or unprimed MSCs. Fold proliferation was calculated relative to proliferation of PBMCs when cultured with stimulator allogenic PBMCs which was considered to be 100. This experiment was performed with different isolates of BMMSCs and WJMSCs and similar results were obtained. One such experiment done in triplicates at passage 5 (P5) is depicted in the table. * *p-* value <0.05 for 1% control MSCs versus 1% TNFα treated MSCs.

### MSCs Derived from Bone Marrow and Wharton's Jelly Modulate Mitogen Induced Lymphoproliferative Responses to Different Extents

WJMSCs and BMMSCs do respond to inflammatory stimuli (Figure 1, 2) and inflammation might influence the immune-modulatory capacity of MSCs. To check this possibility WJMSCs and BMMSCs either primed or unprimed with IFNγ or TNFα, were washed thoroughly and co-cultured with PHA-treated PBMCs at 10% or 1% dosage as described in material and methods. MSCs treated and untreated with mitomycin C were used, for co-culture; to replicate the *in vivo* scenario where we expect proliferating MSCs to interact with immune cells. BMMSCs caused a mild suppression of mitogen simulated lympho-proliferation. IFNγ primed BMMSCs as well as TNFα primed BMMSCs were able to cause suppression even at 1% cell dosage (Table 2) at least in 3 of the 4 donor samples studied. However, significant suppression was not observed when mitomycin C treated BMMSCs were used in the co-culture experiments suggesting that the suppression caused was dependent on BMMSC proliferation.

**Table 2:** Mitogen induced lymphoproliferation responses upon co-culture with Bone marrow derived MSCs (BMMSCs).

Fold Proliferation ± Standard deviation				
Co-culture conditions	BMMSC1 (P5)	BMMSC2 (P5)	BMMSC3 (P6)	BMMSC4 (P6)
+10% BMMSC's	*87.02±2.84*	*85.65±10.60*	*85.85±0.78*	*84.39±5.00*
+1% BMMSC's	***97.06±5.92***	***108.87±12.71***	***87.77±1.69***	***98.67±9.61***
+10% IFN-γ Treated BMMSC's	*67.50±3.96*	*77.63±12.71*	*79.34±2.08*	*78.01±0.74*
+1% IFN-γ Treated BMMSC's	***84.77±3.65***	***97.43±6.60***	***70.54±5.54***	***80.69±1.06***
+10% TNF-α Treated BMMSC's	*80.81±2.15*	*73.25±7.10*	*69.62±2.36*	*82.25±1.94*
+1% TNF-α Treated BMMSC's	83.67±3.58	133.06±10.29	72.54±9.19	82.30±1.77
+10% BMMSC's#	103.01±1.03	121.93±21.61	81.09±5.71	104.48±6.88
+1% BMMSC's#	113.87±3.05	102.95±12.61	83.63±2.05	98.61±1.33
+10% IFN-γ Treated BMMSC's#	87.12±5.88	94.96±17.76	80.57±3.51	133.33±3.80
+1% IFN-γ Treated BMMSC's#	115.33±2.04	125.84±13.39	86.25±5.66	107.29±1.59
+10%TNF-αTreated BMMSC's#	101.81±3.43	75.36±6.83	74.56±4.42	123.41±6.01
+1% TNF-α Treated BMMSC's#	114.25±3.73	119.67±14.09	76.48±4.38	127.17±14.21

P is the passage no of MSC used. “+” in the co-culture conditions indicates fold proliferation in conditions were PHA-treated PBMCs were co-cultured with the Mitomycin treated# or untreated BMMSCs either unprimed or primed with IFNγ or TNFα. Differently treated BMMSCs (as mentioned above) were co-cultured with PHA stimulated PBMCs for 80 hrs and BrdU incorporation was measured subsequently. Fold Proliferation was calculated with respect to the PHA treated PBMC control which was considered to be 100. Each experiment with individual donors was performed in triplicates and the fold proliferation ± S.D is depicted in the table. * *p*<0.05, ** *p*<0.01; 10% BMMSCs versus 10% IFNγ treated BMMSCs *, 10% BMMSCs versus 10% TNFα treated BMMSCs *, 1% BMMSCs versus 1% IFNγ treated BMMSCs **, 1% WJMSCs versus 1% BMMSCs *.

Wharton's jelly derived MSCs suppressed mitogen induced lympho-proliferation responses to a greater extent then bone marrow derived MSCs (Table 2, Table 3 and Table S2). In contrast to BMMSCs, suppression of lymphoproliferation responses were noticed even in 1% WJMSC co-cultures (*p-* value <0.05 was obtained when co-cultures of BMMSCs and WJMSCs were compared). IFNγ or TNFα primed WJMSCs did not further enhance the suppressive responses drastically as compared to the untreated WJMSC. However, the suppression again seemed to be dependent on the proliferative status of WJMSCs. Thus it looks like priming with inflammatory stimuli can enhance the ability of only BMMSCs and not WJMSCs to suppress mitogen induced lymphoproliferation.

**Table 3:** Mitogen induced lymphoproliferation responses upon co-culture with Wharton's jelly derived MSCs (WJMSCs).

	Fold Proliferation ± Standard deviation			
Co-culture conditions	WJMSC 1 (P3)	WJMSC2(P4)	WJMSC3(P5)	WJMSC4(P6)
+10% WJMSC's	97.76±8.52	51.38±10.20	55.0±2.2	50.93±0.64
+1% WJMSC's	*84.00±9.58*	*49.76 ±1.34*	*84.4±6.9*	*50.27±2.88*
+10% IFN-γ Treated WJMSC's	62.05±0.38	37.05±16.78	54.0±3.8	63.38±2.65
+1% IFN-γ Treated WJMSC's	70.19 ±3.63	49.76±11.84	58.1±7.6	54.94±1.94
+10% TNF-α Treated WJMSC's	63.79±4.92	61.27±7.29	57.4±5.9	58.79±6.05
+1% TNF-α Treated WJMSC's	77.06±0.1	95.39±6.66	58.9±4.3	53.24±0.73
+10% WJMSC's#	94.30±5.43	113.86±22.70	82.1±15.5	67.44±7.55
+1% WJMSC's#	87.57±12.27	102.13±16.56	94.9±3.1	81.91±5.48
+10% IFN-γ Treated WJMSC's#	64.40±0.23	121.85±32.05	72.9±2.2	69.27±5.79
+1% IFN-γ Treated WJMSC's#	86.53±0.62	124.05±49.30	102.5±3.6	78.82±3.39
10% TNF-α Treated WJMSC's#	90.35±4.62	100.05±13.66	79.6±3.6	68.03±2.73
1% TNF-α Treated WJMSC's#	80.72±5.40	126.97±9.49	105.4±5.7	79.56±1.67

P is the passage no of MSC used. “+” in the co-culture conditions indicates fold proliferation in conditions were PHA-treated PBMCs were co-cultured with the Mitomycin treated# or untreated WJMSCs either unprimed or primed with IFNγ or TNFα. Differently treated WJMSCs (as mentioned above) were co-cultured with PHA stimulated PBMCs for 80 hrs and BrdU incorporation was measured subsequently. Fold Proliferation was calculated with respect to the PHA treated PBMC control which was considered to be 100. Each experiment with individual donors was performed in triplicates and the fold proliferation ± S.D is depicted in the table. * *p*<0.05, 1% WJMSCs versus 1% BMMSCs.

### BMMSCs and WJMSCs Attenuate Allogenic Mixed Lymphocyte Responses to Different Extents

Different doses of primed or unprimed MSCs were added to MLRs containing PBMCs as responders and mitomycin treated allogenic PBMCs as stimulators. This mimics the *in vivo* GVHD reaction where MSCs are used as third party cells. BrdU incorporation was measured after 6 days. 10% unprimed or primed BMMSCs exhibited comparable suppression on MLRs. Lower dosage (1%) of primed and unprimed BMMSCs in the cultures resulted in an increase of proliferation in few of the samples tested (Table 4).

**Table 4:** Effect of BMMSCs on MLR responses.

	Fold Proliferation ± Standard deviation			
Co-culture conditions	BMMSC1 (P5)	BMMSC2 (P5)	BMMSC3 (P6)	BMMSC4 (P6)
+10% BMMSC's	*50.73±5.6*	*64.58±15.28*	*82.76±3.58*	*73.54±6.97*
+1% BMMSC's	95.00±6.6	170.36±29.02	115.35±3.87	105.83±4.27
+10% IFN-γ Treated BMMSC's	57.86±5.97	70.75±38.85	73.32±5.83	68.57±5.64
+1% IFN-γ Treated BMMSC's	80.11±13.25	198.01±18.46	108.56±2.11	75.98±7.01
10% TNF-α Treated BMMSC's	43.69±9.58	58.66±5.50	79.09±0.45	85.09±8.65
1% TNF-α Treated BMMSC's	94.20±11.66	268.27±14.45	112.35±2.87	84.68±3.93

P is the passage no of BMMSC used. BMMSCs treated or untreated with IFNγ or TNFα were co-cultured with mismatched responder and stimulator PBMCs and the allogenic response generated was measured by BrdU incorporation after 6 days in culture. Each experiment was performed in triplicates. Fold proliferation was calculated relative to proliferation rates of responder PBMCs in the absence of MSCs which was considered to be 100. * *p*<0.05, 10% WJMSCs versus 10% BMMSCs.

Drastic suppression of MLR responses was observed with WJMSCs as compared to BMMSCs (*p-*value 0.01). As compared to BMMSCs the suppressive effect was seen even at 1% dose ratio of primed and unprimed WJMSCs. Suppression was significantly more when 10% IFNγ treated cells were added to the MLRs (Table 5) suggesting that probably IFNγ treated WJMSCs are more potent immune-modulators of allogenic responses (*p*<0.01).

**Table 5:** Effect of WJMSCs on MLR responses.

	Fold Proliferation ± Standard deviation			
Co-culture conditions	WJMSC1(P4)	WJMSC2(P5)	WJMSC3(P6)	WJMSC (P4)
+10% WJMSC's	***47.48±10.39***	***21.0±14.0***	***32.05±8.36***	***42.10±12.33***
+1% WJMSC's	78.36±24.07	92.7±9.8	60.08±5.57	54.66±3.21
+10% IFN-γ Treated WJMSC's	***29.78±4.22***	***16.4±1.1***	***10.31±4.46***	***26.62±8.08***
+1% IFN-γ Treated WJMSC's	81.78±6.55	103.2±2.0	69.98±2.44	51.64±12.24
10% TNF-α Treated WJMSC's	53.57±16.23	31.3±27.0	28.61±2.03	42.60±6.42
1% TNF-α Treated WJMSC's	60.29±7.72	48.8±3.6	56.59±3.64	40.90±0.95

P is the passage no of WJMSC used. WJMSCs treated or untreated with IFNγ or TNFα were co-cultured with mismatched responder and stimulator PBMCs and the allogenic response generated was measured by BrdU incorporation after 6 days in culture. Each experiment was performed in triplicates. Fold proliferation was calculated relative to proliferation rates of responder PBMCs in the absence of MSCs which was considered to be 100. * *p*<0.05, 10% WJMSCs versus 10% BMMSCs, ** *p*<0.01, 10% WJMSCs versus 10% IFNγ treated WJMSCs.

### Cytokine Secretion Profiles of Activated PBMCs in Co-Cultures with Primed MSCs

**Figure 3:**
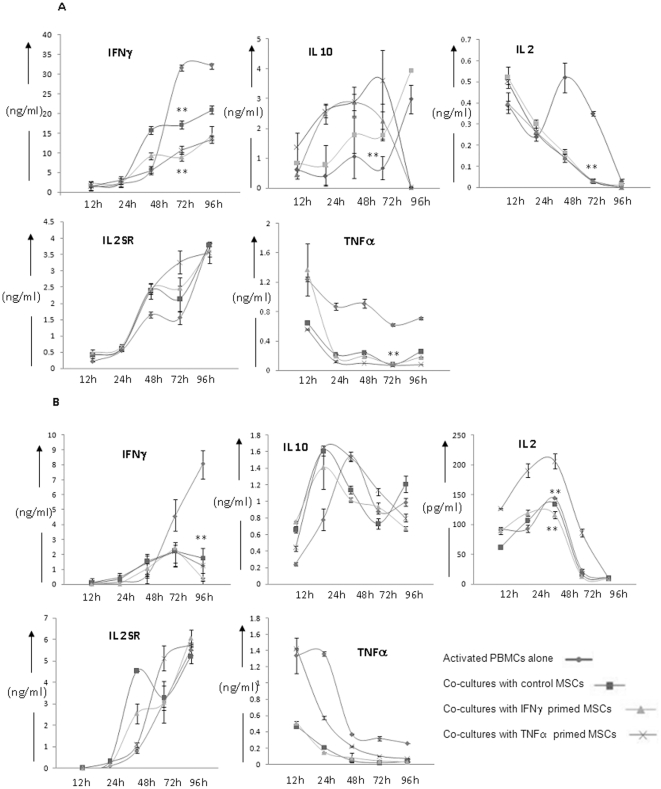
Cytokine secretions from activated lymphocytes are differentially altered upon co-culture with BMMSCs and WJMSCs. The graphs represent amounts of cytokine released (y axis) from MSC/PHA-activated PBMC co-cultures at different time points of activation (x axis). Panel A and Panel B refers to cytokine secretions in BMMSC and WJMSC co-cultures respectively.

To analyze whether MSCs affect the cytokine secretion profiles of activated lymphocytes WJMSCs and BMMSCs were co-cultured with PHA-treated PBMCs for different time intervals and the supernatants were collected. Supernatants were evaluated for key immune-modulating cytokines. BMMSCs and WJMSCs resulted in reduction in secreted levels of IFNγ in a dose dependent manner in activated PBMC cultures (Figure 3). Primed BMMSCs caused a greater reduction in IFNγ than untreated BMMSCs (*p*<0.01). Co-cultures with BMMSCs resulted in a minor increase in IL-10 levels as compared to the control samples. However, a significant increase in IL-10 levels was observed in co-cultures with either IFNγ or TNFα primed BMMSCs consistent with higher reduction in IFNγ levels (*p*<0.05). Only a marginal increase in IL-10 levels was noticed with WJMSC at earlier time points.

A peak in IL-2 levels was observed in PHA stimulated lymphocytes at 48 hr. BMMSCs changed the IL-2 kinetics and secretion levels of activated lymphocytes. A consistent decrease in IL-2 was observed beyond 24 hr as compared to the control PHA activated PBMC cultures. WJMSCs did not cause a significant change in the kinetics and threshold levels of IL-2 as compared to the control samples except for the TNF α primed WJMSC co-cultures were higher threshold levels of IL-2 were observed at all the time points studied. Consistently IL-2SR levels increased beyond 48 hr in the PHA activated control samples. Secreted levels of IL-2SR were marginally more in BMMSC co-cultures and WJMSC co-cultures than the control samples at all the earlier time points studied (Figure 3 and Table S3).

No significant change in the secretion levels and kinetics of IL-1β and IL-6 (data not shown) was noticed with either BMMSC or WJMSC co-cultures.

IFNγ produced by lymphocytes can act as a crucial signal for release of TNFα by activated macrophages. Evaluation of TNFα secretion profiles in the co-culture conditions indicated a decrease in the secreted levels with both BMMSCs and WJMSCs consistent with the decrease in IFNγ levels. Thus in a nutshell, WJMSCs and BMMSCs might modify the immune response by inhibiting the secretion of pro-inflammatory cytokines and increasing the levels of suppressive cytokines like IL-10. Importantly, enhanced reduction in pro-inflammatory cytokines like IFNγ and TNFα upon co-culture with primed BMMSCs could explain the increase in suppression of PHA-induced lymphoproliferation in co-cultures with primed BMMSCs.

### Activation Marker Profile on Stimulated Lymphocytes Is Altered by WJMSCs and BMMSCs Differently

**Figure 4:**
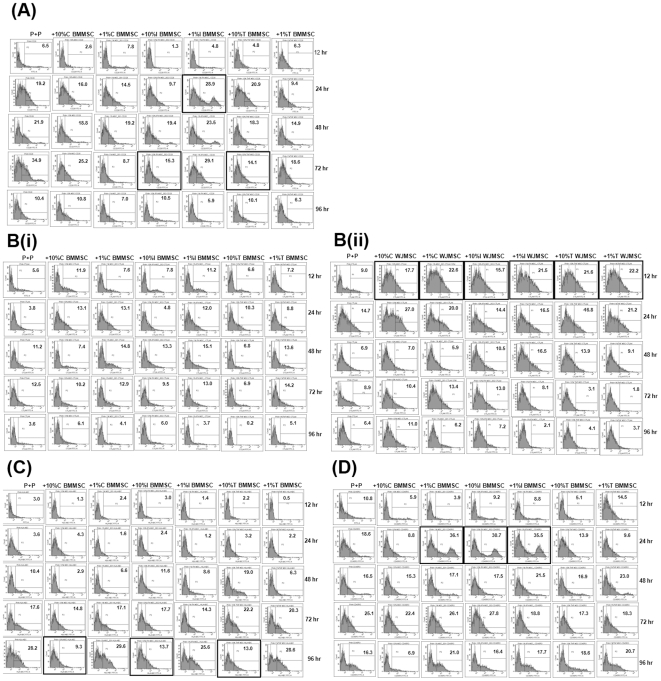
Expression of key activation markers on PHA-stimulated lymphocytes were differentially altered upon co-culture with primed BMMSCs and WJMSCs. Flow cytometry analysis of CD28 (4A), CTLA4 (4B {i}, 2B {ii}), HLA-ABC (4C) and CD45RO (4D) on PHA-treated PBMCs was performed kinetically during different co-culture conditions. “P+P” is PBMC stimulated with PHA and “+” refers to activation markers on PHA-stimulated PBMCs when co-cultured with primed or unprimed MSCs. Key changes are highlighted. % positive cells depicted within each histogram are calculated using FACSDiva software.

MSCs affect the cytokine secretion profiles in mitogen stimulated PBMC cultures. Further, the expression of activation markers on mitogen stimulated PBMCs was evaluated upon co-culture with either primed or unprimed MSCs. Only markers which were obviously altered upon co-culture with MSCs are depicted in Figure 4. The percent positive expression of all the markers studied is depicted in Table 6 and Table 7 in detail.

**Table 6:** Co-culture with BMMSCs derived from bone marrow result in changes in expression patterns of key immune activation markers on stimulated lymphocytes.

	**12 h**	**24 h**	**48 h**	**72 h**	**96 h**		**12 h**	**24 h**	**48 h**	**72 h**	**96 h**
**CD69**						**HLA-ABC**					
**P+P**	1.5	12.2	23.8	28.6	12.5	**P+P**	3.0	3.6	10.4	17.6	28.2
**+10%C**	2.1	11.6	21.2	21.8	5.9	**+10% C**	1.3	4.3	2.9	14.8	9.3
**+1% C**	6.6	0.0	23.5	21.8	6.4	**+1% C**	2.4	1.6	6.6	17.1	29.6
**+10% I**	2.8	9.4	19.7	18.0	11.4	**+10% I**	3.0	2.4	11.6	17.7	13.7
**+1% I**	6.7	11.0	18.1	12.5	5.7	**+1% I**	1.4	1.2	8.6	14.3	25.6
**+10% T**	6.2	15.0	14.1	16.0	4.9	**+10% T**	2.2	3.2	19.0	22.2	13.0
**+1% T**	2.6	11.6	17.4	14.0	13.8	**+1% T**	0.5	2.2	6.3	20.3	28.6
**CD28**						**CD-45RO**					
**P+P**	6.5	19.2	21.9	34.9	10.4	**P+P**	10.8	18.6	16.5	25.1	16.3
**+10% C**	2.6	16.0	18.8	25.2	10.8	**+10% C**	5.9	8.8	15.3	22.4	6.9
**+1% C**	7.8	14.5	19.2	8.7	7.0	**+1% C**	3.9	36.1	17.1	26.1	21.0
**+10% I**	1.3	9.7	19.4	15.3	10.5	**+10% I**	9.2	30.7	17.5	27.8	16.4
**+1% I**	4.8	28.9	23.5	29.1	5.9	**+1% I**	8.8	35.5	21.5	18.8	17.7
**+10% T**	4.8	20.9	18.3	14.1	10.1	**+10% T**	5.1	13.9	16.9	17.3	18.6
**+1% T**	6.3	9.4	14.9	18.6	6.3	**+1% T**	14.5	9.6	23.0	18.3	20.7
**CTLA4**						**CD-45RA**					
**P+P**	5.6	3.8	11.2	12.5	3.6	**P+P**	28.9	11.6	28.5	28.9	37.4
**+10% C**	11.9	13.1	7.4	10.2	6.1	**+10% C**	4.0	8.0	29.4	33.7	19.0
**+1% C**	7.6	13.1	14.8	12.9	4.1	**+1% C**	8.5	11.9	25.4	28.2	26.9
**+10% I**	7.8	4.8	13.3	9.5	6.0	**+10% I**	27.1	11.2	25.7	22.2	32.6
**+1% I**	11.2	12.0	15.1	13.0	3.7	**+1% I**	22.4	11.7	22.2	28.4	22.1
**+10% T**	6.6	10.3	6.8	6.9	0.2	**+10% T**	16.6	6.8	27.8	14.5	24.8
**+1% T**	7.2	8.8	13.6	14.2	5.1	**+1% T**	7.7	12.3	18.5	19.5	36.8

PBMCs were stimulated with PHA in the presence of different dosage of BMMSCs either untreated or treated with inflammatory stimuli IFNγ (I) and TNFα (T) for different time intervals. Subsequently PBMCs were picked and the surface expression of activated PBMCs under different co-culture conditions was monitored by Flow cytometry. % positive cells expressing the specific marker are depicted in the table were obtained using FACSDiva software.

**Table 7:** Co-culture with WJMSCs derived from Wharton's jelly result in changes in expression patterns of key immune activation markers on stimulated lymphocytes.

	**12 h**	**24 h**	**48 h**	**72 h**	**96 h**		**12 h**	**24 h**	**48 h**	**72 h**	**96 h**
CD69						HLA-ABC					
**P+P**	24.5	24.4	5.0	1.6	2.9	**P+P**	26.7	27.4	21.3	32.3	46.7
**+10%C**	16.0	27.0	9.3	4.1	3.8	**+10% C**	30.0	26.1	22.1	32.7	36.8
**+1% C**	21.1	27.4	5.3	5.7	3.5	**+1% C**	32.0	27.6	22.7	38.2	53.4
**+10% I**	22.7	25.9	8.4	5.0	6.4	**+10% I**	32.2	36.5	23.4	40.9	39.6
**+1% I**	13.6	22.4	10.1	4.3	2.5	**+1% I**	32.1	30.5	32.6	31.8	46.6
**+10% T**	7.4	23.5	5.6	3.7	5.3	**+10% T**	37.6	29.8	34.4	37.1	41.6
**+1% T**	19.4	19.0	15.1	7.8	2.3	**+1% T**	29.9	26.0	28.9	24.8	46.9
**CD28**						**CD-45RO**					
**P+P**	22.8	15.7	10.1	2.9	2.4	**P+P**	28.4	24.6	18.1	1.9	12.7
**+10% C**	15.5	16.9	8.3	5.9	4.7	**+10% C**	25.3	30.8	14.8	9.6	14.5
**+1% C**	18.4	24.1	9.7	6.0	4.2	**+1% C**	28.4	28.1	13.9	20.4	34.2
**+10% I**	14.6	18.6	8.1	6.9	6.3	**+10% I**	21.2	22.7	18.1	11.5	16.3
**+1% I**	23.1	18.9	8.4	5.0	3.0	**+1% I**	23.6	29.0	11.7	8.3	17.3
**+10% T**	18.5	16.1	10.6	8.9	3.4	**+10% T**	27.1	21.6	12.3	6.3	18.5
**+1% T**	32.4	20.8	8.0	4.6	1.8	**+1% T**	27.9	24.9	12.2	9.8	10.5
**CTLA4**						**CD-45RA**					
**P+P**	9.0	14.7	6.9	8.9	6.4	**P+P**	34.7	34.5	36.4	9.0	15.3
**+10% C**	17.7	27.0	7.0	10.4	11.0	**+10% C**	26.6	37.1	26.6	24.5	19.4
**+1% C**	22.6	20.0	5.9	13.4	6.2	**+1% C**	41.8	41.8	25.3	20.4	13.6
**+10% I**	15.7	14.4	10.5	13.0	7.2	**+10% I**	29.1	35.0	38.9	16.1	17.1
**+1% I**	21.5	16.5	16.5	8.1	2.1	**+1% I**	38.1	42.7	35.4	12.9	14.7
**+10% T**	21.6	16.8	13.9	3.1	4.1	**+10% T**	28.3	39.0	35.2	20.9	15.0
**+1% T**	22.2	21.2	9.1	1.8	3.7	**+1% T**	30.1	29.5	35.7	12.6	26.2

PBMCs were stimulated with PHA in the presence of different dosage of WJMSCs either untreated or treated with inflammatory stimuli IFNγ (I) and TNFα (T) for different time intervals. Subsequently PBMCs were picked and the surface expression of activated PBMCs under different co-culture conditions was monitored by Flow cytometry. % positive cells expressing the specific marker are depicted in the table were obtained using FACSDiva software.

CD69, an early activation marker on T cells, was not changed upon co-culture with BMMSCs and WJMSCs except for TNFα primed BMMSC and WJMSCs where a reduction in surface levels was observed.

CD28, a positive co-stimulatory ligand on T lymphocytes, was diminished on co-culture with both 10% IFNγ and 10% TNFα primed BMMSCs. Surprisingly, at earlier time points co-culture with 1% IFNγ primed BMMSCs resulted in a sub-population of CD28 high lymphocytes which was not sustained at later time points (Figure 4A). CD28 levels do not seem to be modulated by WJMSCs.

Co-culture with WJMSCs results in a substantial change in the kinetics (expression was seen as early as 12 hr of activation) and expression levels of the negative co-stimulatory ligand, CTLA4 on PBMCs (Figure 4B {ii}). A very mild increase in CTLA4 was observed with BMMSCs but the effect was not as striking as in the case of WJMSCs (Figure 4B {i}). A decrease in MHC class I expression was observed upon co-culture with 10% BMMSCs either primed or unprimed (Figure 4C). The decrease was not so evident with WJMSC co-cultures.

CD45RA and CD45RO markers are used to identify naïve and memory T cell responses depending on their relative expression levels [47]. Co-culture with BMMSCs results in a transient up regulation of CD45RO levels at 24 hr with 1% BMMSCs and with IFNγ primed BMMSCs at both the doses tested (subpopulations of high CD45RO expression were observed, Figure 4D). CD45RO expression pattern did not change much upon WJMSC co-culture. However, no definitive conclusions could be drawn from alterations in CD45RA expression data.

No significant changes in the levels of CD4, CD8, CD95 was observed in the co-culture conditions tested. Annexin V staining was not detected on the activated PBMCs probably suggesting that PBMCs are not undergoing apoptosis on co-culture with either WJMSCs or BMMSCs (data not shown).

BMMSCs and WJMSCs could probably influence lymphoproliferation responses uniquely by differentially modulating the levels of key immune activation markers which contribute to the development of the immune response.

### Differences in Levels of Immune-Suppressive Molecules Expressed by BMMSCs and WJMSCs

**Figure 5:**
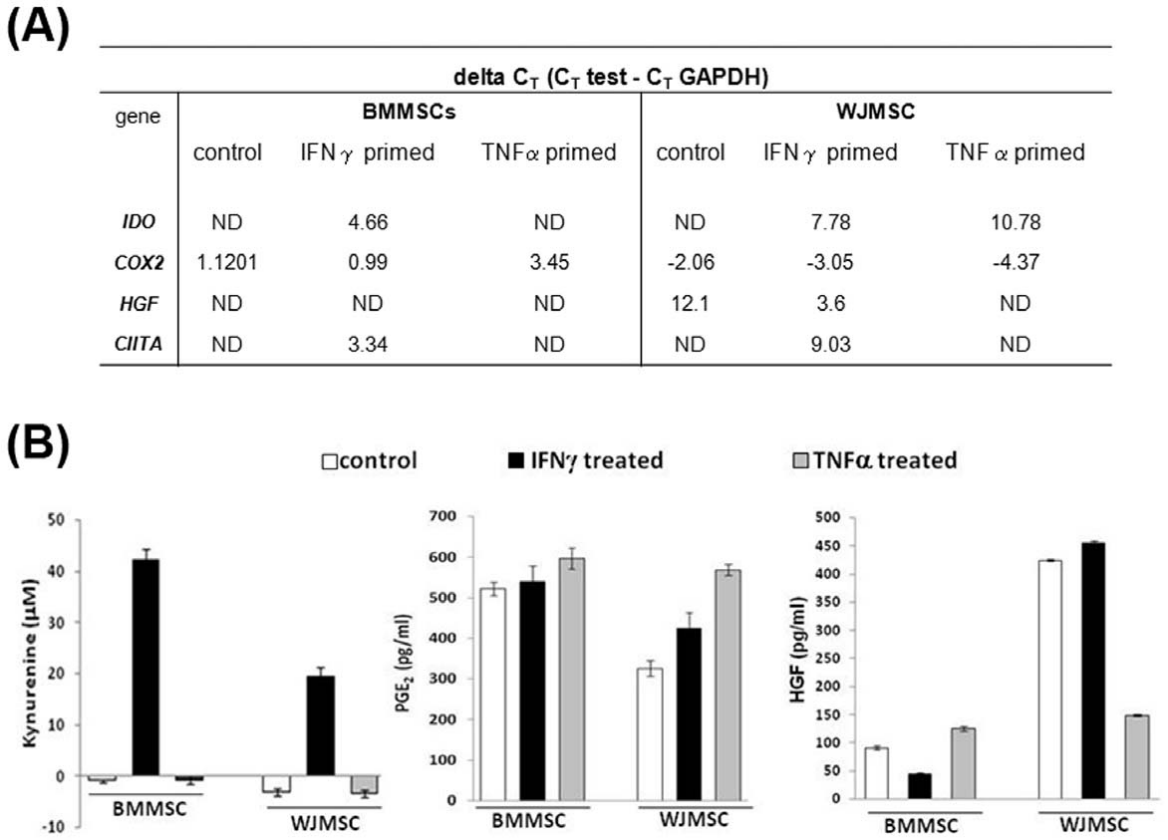
Differences in immune-suppressive gene and protein levels in primed/unprimed BMMSC and WJMSCs. (A) Delta Cycle Threshold (delta C_T_) is obtained after subtracting the endogenous control (GAPDH) Cycle Threshold from the test. ND refers to not detected. (B) IDO activity (reflected by Kynurenine amounts), PGE2 and HGF levels are depicted.

To explore the reason behind the mechanistic differences in immune-modulation observed quantitative RT-PCR analysis was performed to screen for differences in expression of known immune-suppressive factors in primed/unprimed MSCs. Differences in mRNA expression of *IDO*, *HGF*, *COX2* and *CIITA* was consistently observed (Figure 5A). Based on the PCR screen IDO enzyme activity levels and secretion levels of HGF and COX2 generated PGE2 was evaluated in supernatants of primed/unprimed MSCs.

IDO is an IFNγ inducible enzyme which depletes Tryptophan and thus inhibits T cell proliferation [48]. IDO has not been shown to be expressed by MSCs but is inducible by IFNγ. [49]. IFNγ inducible IDO activity was higher in IFNγ primed BMMSCs than IFNγ primed WJMSCs (Figure 5B).

CIITA, the master regulator of MHC class II expression [41] was induced only in IFNγ primed MSCs consistent with the lack of HLA-DR expression in control and TNFα primed MSCs (Figure 2). Lower transcript levels of CIITA was detected in IFNγ primed WJMSCs than BMMSCs. HLA-DR cell surface expression was not detected on IFNγ primed WJMSCs despite the presence of CIITA suggesting a probable regulation at protein levels or very late activation kinetics as previously described [41].

Similarly, BMMSCs secreted higher levels of PGE2 than WJMSCs (Figure 5B). Although TNFα has been shown to induce BMMSC secreted PGE2 in our hands the TNFα induction was not so robust [8]. However, WJMSC secreted PGE2 was induced upon priming with IFNγ and more particularly with TNFα.

A drastic difference in HGF secretion as well as transcript levels was observed in both the MSCs (Figure 5A, B). TNFα priming of WJMSCs resulted in marked reduction of HGF secretion.

Taken together the present data emphasizes differences in immune-suppressive factors between BMMSCs and WJMSCs and their induction with pro-inflammatory cytokines.

## Discussion

Bone marrow and Wharton's jelly derived MSCs were evaluated for expression of immunologically relevant ligands upon treatment with IFNγ and TNFα. Both BMMSCs and WJMSCs seemed to have a hypo-immune profile, as evidenced by lack of co-stimulatory ligands, CD80/CD86 and CD50 even on pretreatment with IFNγ and TNFα. HLA-ABC and immuno-adhesive ligand, CD54 was greatly induced by IFNγ and TNFα. HLA-DR was induced only in BMMSCs on IFNγ treatment and not so in WJMSCs (Figure 2). Although HLA-DR has been reported to be greatly induced by IFNγ in both WJMSCs [50] and BMMSCs [46] HLA-DR was only mildly induced in WJMSCs as compared to BMMSCs in our hands. It's possible that WJMSCs and BMMSCs respond to different thresholds of inflammation. Although induction of MHC class I and Class II was observed on IFNγ treatment, no co-stimulatory molecules were detected, suggesting a tolerogenic phenotype even under inflammatory conditions [46]. Our observations are in coherence with these results. In addition we suggest that probably minor differences do exist in the way different MSCs respond to different kinds and thresholds of inflammatory stimuli. Pretreatment with IFNγ and TNFα resulted in changes in the morphology and growth characteristics of BMMSCs (Figure 1A, Figure S4). However, markers reported to be present on mesenchymal cells SSEA4 and CD73 (Figure 2) did not change drastically on priming with either IFNγ or TNFα Differentiation capacity of MSCs to all the three mesenchymal lineages was also not compromised after priming (Figure 1B,C,D). Thus pretreatment with inflammatory cytokines does not affect the “mesenchymalness” of WJMSCs and BMMSCs.

There is lot of confusion in the literature regarding the immunogenicity of MSCs in an allogenic platform [19], [51], [52]. MSCs do not express blood group antigens and no alloantibodies were detected post MSC transplantation in allogenic Hematopoietic stem cell transplantation (HSCT) recipients [53]. Weiss *et al.*, 2008 has shown that WJMSCs do not elicit allogenic as well as Xenogenic responses *in vitro* [29]. However, intracardiac allogenic porcine MSCs have shown to elicit an immune response contradicting the *in vitro* data [54]. Porcine umbilical cord derived stem cells did not induce a considerable immune response *in vivo* but stimulation with IFNγ or injection in an inflamed region resulted in immunogenicity [50]. These discrepancies could be partly due to heterogeneous MSC populations when isolated from different tissues or the influence of environmental factors like inflammation affecting the immunogenicity of MSCs. Thus the immunogenicity of both WJMSCs and BMMSCs were evaluated after priming with pro-inflammatory cytokines in a one way MLR (Table 1). Although significantly high immunogenicity was not observed in different pre-treatment conditions, TNFα treatment of WJMSCs resulted in slight enhancement of immunogenicity (Table 1) suggesting that different pro-inflammatory cytokines can exert different effects in conferring immunogenicity to MSCs depending upon the predominant cytokine at the site of inflammation. Low dose MSCs seems to elicit a lower proliferative response in PBMCs as compared to PBMC alone control within the limitations of the assay sensitivity (Table 1). There is enough evidence which now suggests that MSCs are not inherently immune-evasive and the immune-privilege status is a flexible state depending upon cell to cell interactions and importantly soluble factors secreted by MSCs [35]. Fange *et al.*, 2006 have elegantly shown the importance of cells numbers in affecting allo-T cell proliferation [55].The report clearly suggests that depending on the extent of immune suppressive effects and allo-stimulatory potential of MSCs the balance can mildly shift towards either higher suppression or low alloreactivity. Data depicted in Table 1 indicates only very mild allo-reactivity to MSCs (10% dosage) in comparison to allogenic PBMCs which is considered as 100%. It's possible that at 1% dosage the suppressive mechanisms overrule the mild allo-reactivity in our case.

WJMSCs and BMMSCs pretreated with IFNγ and ΤΝFα modulated mitogen induced and allo-antigen induced lymphoproliferation responses distinctly (Table 2, Table 3, Table 4, and Table 5). WJMSCs were more suppressive than BMMSCs as evident by suppression observed even at lower dosage. In addition, inflammation seemed to enhance the capability of BMMSCs to attenuate PHA induced lymphoproliferation but not of WJMSCs (Table 2, Table 3 and Figure S6). WJMSCs attenuated MLR reactions to a greater extent than BMMSCs (Table 4 and Table 5). In comparison to mitogen-induced lymphoproliferation, BMMSCs seemed to cause a more prominent suppression of MLR. Primed BMMSCs are not better in suppressing MLRs than unprimed BMMSCs. In contrast to BMMSCs, priming of WJMSCs by IFNγ seems to marginally enhance their capacity to attenuate MLRs. All together, it appears that mitogen induced lymphoproliferation and allo-antigen driven lymphoproliferation are modulated probably by different mechanisms unique for BMMSCs and WJMSCs. In a previous report it was shown that fetal human MSCs inhibit proliferation to PHA and not to alloresponses in MLRs [32]; however these responses were not evaluated in comparison to BMMSCs. The differences observed with PHA-induced and alloantigen driven lymphoproliferation responses in different conditions might be due to the fact that in a polyclonal setting MSCs inhibit activation of distinct T cells. In a more complex immune reaction like a MLR, the immune-suppressive effect on both the PBMCs by MSCs as well as the weak allogeneic response against the primed/unprimed MSC must be considered. Even mild immunogenicity conferred to MSCs upon priming could attribute to the differential effects (Table 1). Thus exposure to inflammatory stimuli might even influence the immune-regulatory behavior of WJMCS and BMMSCs in a distinct fashion.

To understand the differences observed in the extent of immune-suppression caused by BMMSCs and WJMSCs, we probed into the cytokine secretion profiles of PHA-activated lymphocytes in co-cultures with either primed or unprimed MSCs. Both BMMSCs and WJMSCs reduce the production of pro-inflammatory cytokines IFNγ and TNFα (Figure 3 and Table S3). However, the kinetics and threshold levels of pro-inflammatory cytokine IL-2 was modulated only by BMMSCs and not with WJMSCs except when primed with TNFα. BMMSCs but not WJMSCs enhanced the levels of immune suppressive cytokine IL-10. Taken together although both MSCs skew the cytokine secretion profiles from a pro-inflammatory to an anti-inflammatory phenotype, in depth analysis indicate differences in the type of cytokines modulated by both the MSCs studied. The differences in the cytokine profiles in lymphoproliferation reactions under different co-culture conditions could account for the differences in extent of immune-suppression observed with primed BMMSCs and WJMSCs.

Lymphocyte proliferation is marked by characteristic signatures of activation marker expression which eventually modulate the course of the T cell effector response. A combined evaluation of proliferation as well as the time course of activation marker expression would give a clearer insight into the mechanisms of immune modulation [56]. Optimal T cell activation *in vivo* also requires two signals, one being induced by MHC-TCR interaction and the other provided by positive co-stimulatory ligands like CD28. Eventually there is an up-regulation of negative co-stimulatory ligands like CTLA4 which attenuate T cell responses. High CTLA4 levels early on during an activation response have been implicated in tolerance induction [57]. Both antigenic as well as mitogenic stimuli initiate the transition from a CD45RA phenotype to different transitional states of CD45RO positivity and this response is finely tuned by cytokine milieu at the site of inflammation [58].

To substantiate the previous experiments, changes were also observed in cell surface marker profile on mitogen activated PBMCs differentially upon co-culture with primed and unprimed WJMSCs and BMMSCs (Figure 4, Table 6 and Table 7). Priming with either TNFα or IFNγ also caused differential modulation in the surface expression of activated lymphocytes suggesting that these two cytokines could possibly modulate the immune regulatory properties of MSCs differentially. Similar to our observations immune-modulatory effects of murine BMMSCs have been reported to be differentially modulated by IFNγ and TNFα [40]. MSCs could result in suppression by decreasing the levels of positive co-stimulatory markers and increase in negative co-stimulatory molecules on T cells resulting in an anergic state.

Consistent with enhanced suppression with pro-inflammatory cytokine primed BMMSCs a decrease in levels of CD28 was observed at 72 hr of activation (Figure 2A). In case of WJMSCs although positive co-stimulatory ligands were not majorly modulated in comparison to BMMSCs, the very early (12 hr of activation response) and enhanced appearance of negative co-stimulatory ligands like CTLA4 might have resulted in greater suppression despite the presence of CD28 (Figure 4B {ii}). Further a difference in the relative levels of CD45RA and CD45RO was observed only upon co-culture with BMMSCs. Low amounts of BMMSCs (1% dosage) and IFNγ primed BMMSCs only caused an increase in the CD45RO subset at 24 hr of activation suggesting induction of memory like phenotype (Figure 4D) It is possible that low levels of MSCs and priming could result in an initial activation response which is not sustained later on. Current reports in the field suggest that immune suppression by MSCs is not inherent but is acquired upon interaction with immune cells and their secretions [35]. It's possible that MSCs are initially detected by immune cells but later adopts mechanisms to evade the response. Work is ongoing to evaluate in detail the lymphocyte subsets which arise upon interaction with WJMSCs and BMMSCs.

Changes in the activation marker profiles with primed MSCs at different dosages indicate that the overall contribution of MSCs in modulating the signal strength of a T cell response should carefully be evaluated in context of other immune populations before drawing definitive conclusions. Priming with inflammatory stimuli changes the threshold levels of immune adhesive ligands like CD54 (Figure 2) and might also change the cytokine secretion profiles of MSCs which have immunological relevance. Differences in cytokine secretion profiles of BMMSCs and fetal MSCs have been reported [31], [59], [60]. Both soluble and contact-dependent mechanisms have been implicated in MSC immune-modulation [8], [33], [60], [61]. Immune-suppression studies with conditioned media from BMMSCs as well as with culture inserts emphasize on a major role of soluble factors in this phenomenon [8]. Expression levels of known MSC immune-modulatory molecules such as IDO, HGF and COX2 generated PGE2 differed in culture supernatants of both the MSCs (Figure 5). Further these factors were differently modulated by inflammatory stimuli thus suggesting the possibility that inflammation can fine tune the immune properties of various tissue derived MSCs distinctly. Further work is ongoing to definitively dissect out the role of immunosuppressive molecules differentially expressed in both the MSCs in lymphocyte/MSC co-cultures.

In a nutshell, this work emphasizes on the role of local inflammatory factors in modulating the immune-regulatory properties of two different MSCs. MSCs do respond to inflammatory stimuli, taking part actively in an immune response and cannot be just thought of as silent spectators. Lower dosage of cells resulted in slight enhancement in the proliferation rate especially in MLR reactions with BMMSCs (Table 4) and also resulted in mild changes in the activation marker profile of PHA-stimulated PBMCs. Importantly different activation markers and cytokines were modulated by BMMSCs and WJMSCs under different priming conditions. These results indicate that caution should be taken while determining the dosage of MSCs and understanding the pathophysiology of the disease before putting them for therapeutic usage. A recent study by Magatti *et al.*, 2008, indicates the existence of cells with both immune suppression and immune stimulation capabilities within amniotic mesenchymal MSCs [62]. As an off shoot of this work it will be interesting to investigate whether different subsets of MSCs having distinct immune behaviors exist within the heterogeneous pool of MSCs in different tissues. It is possible that these subsets respond differently to inflammatory stimuli. In totality the present work pinpoints and strengthens the recent evidences regarding the inherent differences between MSCs differing in their primitiveness. Stromal cells from different tissues have been shown to exhibit site-specific molecular identity and memory [63]. However there is not enough evidence in the literature which suggests that these affect the immune properties of stromal MSCs. Observations discussed suggest that inherent differences between different MSCs can impact the mechanism and extent of immune-modulation exhibited probably due to differences in immuno-suppressive factor expression and secretion. The hallmark of this study is the identification of putative mechanisms by which different MSCs cause immune-modulation. This work emphasizes that effect of predominant inflammatory cytokines at the site of transplantation should be considered for accessing the immunogenicity and immune-modulatory behavior of MSCs in different clinical settings.

## Supporting Information

Table S1 List of primer sequences used for real-time RT-PCR analysis.
(0.03 MB DOC)

Table S2 Mitogen induced lymphoproliferation responses upon co-culture with bone marrow derived MSCs (BMMSCs) and Wharton's jelly derived MSCs (WJMSCs).
(0.04 MB DOC)

Table S3 MSCs from bone marrow (A) and Wharton's jelly (B) modulate the kinetics and secretion profiles of key cytokines in lymphoproliferation experiments.
(0.14 MB DOC)

Figure S1 WJMSCs and BMMSCs exhibit conserved mesenchymal marker expression (A) and differentiation capacities (B, C). Mesenchymal marker expression at passage 5 is depicted. % positivity for each marker is shown in the figure and isotype control has been used for gating. ND-No positive population detected. (B) Represents Oil O Red staining of adipocytes induced from MSCs (magnification 10×40). (C) Represents Von Kossa staining of mineralized deposits after osteogenic induction of MSCs (magnification 10×10).
(2.86 MB TIF)

Figure S2 Kinetics of expression of HLA-ABC and HLA-DR on MSCs upon treatment with pro-inflammatory cytokines IFNγ and TNFα. BMMSCs (A) and WJMSCs (B) were treated or untreated (black line) with 150 U/ml of IFNγ or 10 ng/ml of TNFα for either 24 hr (grey lines), 48 hr (black dotted lines) or 72 hr (thick black line) and subsequently cell surface levels of HLA-ABC or HLA-DR was evaluated by Flow cytometry. Grey filled histogram represents staining with the matched isotype controls.
(0.61 MB TIF)

Figure S3 BrdU uptake of MSCs treated with Mitomycin C (10μg/ml) is comparable or lower than resting PBMCs in culture. The grey filled histogram represents BMMSCs whereas black filled histogram represents WJMSCs. RFU is relative fluorescence units after subtraction from endogenous fluorescence controls (-BrdU sample).
(1.43 MB TIF)

Figure S4 Exposure to IFNγ and TNFα alters the replication potential of BMMSCs. PD refers to population doublings calculated after priming with either IFNγ or TNFα at each passage (P) as indicated on the x-axis.
(0.49 MB TIF)

Figure S5 Normal karyotype of unprimed and primed WJMSCs and BMMSCs. Karyotype of Passage 5 MSCs is depicted.
(0.73 MB TIF)

Figure S6 BMMSCs and WJMSCs retain their immuno-modulatory properties on exposure to varying doses of pro-inflammatory cytokines. C refers to proliferation in PHA treated PBMCs. “+” refers to co-cultures of PHA treated PBMCs and MSCs primed with different concentrations of IFNγ or TNFα. Representative experiment performed in triplicates is depicted above.
(0.61 MB TIF)
