# Supplementary material for: Pro-Inflammatory Cytokines, IFNγ and TNFα, Influence Immune Properties of Human Bone Marrow and Wharton Jelly Mesenchymal Stem Cells Differentially
Source: PLoS One. 2010 Feb 2;5(2):e9016. doi: 10.1371/journal.pone.0009016 (PMC2814860; doi:10.1371/journal.pone.0009016)
Supplement: Table S1 — List of primer sequences used for real-time RT-PCR analysis. (0.03 MB DOC) [file pone.0009016.s001.doc]

**Table S1**

List of primer sequences used for real-time RT-PCR analysis

| **Gene** | **Forward primer (5’ to 3’)** | **Reverse primer (5’ to 3’)** | **Annealing temperature (^0^C)** |
| --- | --- | --- | --- |
| *IDO1* | CCTGAGGAGCTACCATCTGC | TCAGTGCCTCCAGTTCCTTT | 60 |
| *HGF* | CAATAGCATGTCAAGTGGAG | CTGTGTTCGTGTGGTATCAT | 55 |
| *COX2* | TTCAAATGAGATTGTGGGAAAATTGCT | AGATCATCTCTGCCTGAGTATCTT | 65 |
| *GAPDH* | AGCCACATCGCTCAGACACC | GTACTCAGCGGCCAGCATCG | 60 |
| *CIITA* | CCGACACAGACACCATCAAC | CTTTTCTGCCCAACTTCTGC | 60 |
