# Supplementary material for: Pro-Inflammatory Cytokines, IFNγ and TNFα, Influence Immune Properties of Human Bone Marrow and Wharton Jelly Mesenchymal Stem Cells Differentially
Source: PLoS One. 2010 Feb 2;5(2):e9016. doi: 10.1371/journal.pone.0009016 (PMC2814860; doi:10.1371/journal.pone.0009016)
Supplement: Table S2 — Mitogen induced lymphoproliferation responses upon co-culture with bone marrow derived MSCs (BMMSCs) and Wharton's jelly derived MSCs (WJMSCs). (0.04 MB DOC) [file pone.0009016.s002.doc]

**Table S2**

Mitogen induced lymphoproliferation responses upon co-culture with bone marrow derived MSCs (BMMSCs) and Wharton’s jelly derived MSCs (WJMSCs)

| **Fold Proliferation ± Standard deviation** | | | |
| --- | --- | --- | --- |
| **Co-culture conditions**  +10% BMMSC | **BMMSC5 (P5)**  66.35±2.97 | +10% WJMSC | **WJMSC5(P5)**  31.9 ±3.3 |
| +10% IFN-γ treated BMMSC | 23.84±3.20 | +10% IFN-γ treated WJMSC | 36.1 ±0.8 |
| +10% TNF-α treated BMMSC | 62.90±1.96 | +10% TNF-α treated WJMSC | 42.7 ±7.3 |

P is the passage no of MSC used. “+” in the co-culture conditions indicates fold proliferation in conditions were PHA-treated PBMCs were co-cultured with untreated MSCs either unprimed or primed with IFNγ or TNFα.
