# Supplementary material for: Pro-Inflammatory Cytokines, IFNγ and TNFα, Influence Immune Properties of Human Bone Marrow and Wharton Jelly Mesenchymal Stem Cells Differentially
Source: PLoS One. 2010 Feb 2;5(2):e9016. doi: 10.1371/journal.pone.0009016 (PMC2814860; doi:10.1371/journal.pone.0009016)
Supplement: Table S3 — MSCs from bone marrow (A) and Wharton's jelly (B) modulate the kinetics and secretion profiles of key cytokines in lymphoproliferation experiments. (0.14 MB DOC) [file pone.0009016.s003.doc]

**Table S3**

MSCs from bone marrow (A) and Wharton’s jelly (B) modulate the kinetics and secretion profiles of key cytokines in lymphoproliferation experiments

| **IFNγ (ng/ml)** | | | | |  |
| --- | --- | --- | --- | --- | --- |
| Time(hr) | 12 | 24 | 48 | 72 | 96 |
| **P+PHA** | 1.41±0.25 | 2.17±0.47 | 5.79±0.09 | 31.74±0.62 | 32.36±0.34 |
| **+10% C** | 1.63±0.14 | 2.49±0.49 | 15.76±1.03 | 17.19±2.20 | 20.93±3.20 |
| **+1% C** | 1.51±0.48 | 2.87±0.09 | 17.17±0.77 | 28.06±1.04 | 30.90±0.23 |
| **+10% I** | 2.32±0.49 | 2.46±0.38 | 9.28±0.77 | 8.99±1.07 | 14.37±2.38 |
| **+1% I** | 2.58±1.47 | 2.11±0.37 | 12.54±0.71 | 25.36±1.48 | 31.29±0.54 |
| **+10% T** | 1.55±0.27 | 3.04±0.33 | 5.53±0.89 | 10.72±0.99 | 13.41±1.60 |
| **+1% T** | 1.38±0.38 | 2.28±1.02 | 8.78±1.04 | 27.56±1.49 | 32.69±0.35 |

|  |  | **IL-10 (ng/ml)** | |  |  |
| --- | --- | --- | --- | --- | --- |
| Time(hr) | 12 | 24 | 48 | 72 | 96 |
| **P+PHA** | 0.62±0.21 | 0.40±0.32 | 1.05±0.71 | 0.67±0.39 | 2.98±0.47 |
| **+10% C** | 0.85±0.05 | 0.77±0.66 | 1.77±0.60 | 1.78±0.19 | 3.95±0.03 |
| **+1% C** | 0.89±0.31 | 1.28±0.05 | 1.50±0.59 | 1.41±021 | 0.02±0.03 |
| **+10% I** | 0.45±0.14 | 2.49±0.34 | 2.88±0.27 | 2.24±0.58 | 0.02±0.03 |
| **+1% I** | 0.70±0.44 | 1.14±0.06 | 1.54±0.23 | 1.18±0.18 | 0.03±0.06 |
| **+10% T** | 1.35±0.49 | 2.58±0.20 | 2.91±0.49 | 3.59±1.03 | 0.01±0.02 |
| **+1% T** | 0.50±0.12 | 1.35±0.08 | 1.52±0.30 | 1.12±0.44 | 0.02±0.03 |

|  |  |  | **IL2(ng/ml)** |  |  |
| --- | --- | --- | --- | --- | --- |
| Time (hr) | 12 | 24 | 48 | 72 | 96 |
| **P+P** | 0.39±0.02 | 0.24±0.02 | 0.52±0.07 | 0.35±0.01 | 0.03±0.01 |
| **+10% C** | 0.52±0.05 | 0.30±0.02 | 0.16±0.02 | 0.03±0.01 | 0.01±0.03 |
| **+1% C** | 0.43±0.02 | 0.23±0.01 | 0.24±0.05 | 0.13±0.02 | 0.01±0.01 |
| **+10% I** | 0.40±0.05 | 0.26±0.01 | 0.14±0.02 | 0.03±0.01 | ND |
| **+1% I** | 0.41±0.05 | 0.19±0.01 | 0.23±0.06 | 0.13±0.01 | 0.01±0.01 |
| **+10% T** | 0.50±0.01 | 0.26±0.02 | 0.14±0.02 | 0.03±0.01 | ND |
| **+1% T** | 0.46±0.02 | 0.23±0.00 | 0.37±0.04 | 0.22±0.01 | 0.05±0.01 |

|  |  | **IL-2sR(ng/ml)** | |  |  |
| --- | --- | --- | --- | --- | --- |
| Time(hr) | 12 | 24 | 48 | 72 | 96 |
| **P+P** | 0.21±0.01 | 0.54±0.02 | 1.64±0.09 | 1.56±0.20 | 3.84±0.01 |
| **+10% C** | 0.42±0.06 | 0.60±0.03 | 2.39±0.08 | 2.14±0.40 | 3.79±0.04 |
| **+1% C** | 0.29±0.10 | 0.39±0.34 | 2.20±0.14 | 2.09±0.31 | 3.74±0.10 |
| **+10% I** | 0.48±0.11 | 0.67±0.07 | 2.44±0.16 | 2.47±0.33 | 3.64±0.21 |
| **+1% I** | 0.43±0.09 | 0.36±0.14 | 2.02±0.23 | 2.74±0.44 | 3.89±0.02 |
| **+10% T** | 0.40±0.10 | 0.62±0.09 | 2.38±0.24 | 3.26±0.35 | 3.56±0.33 |
| **+1% T** | 0.40±0.07 | 0.50±0.04 | 1.72±0.16 | 2.90±0.08 | 2.98±1.52 |

|  | **TNFα (ng/ml)** | | | | |
| --- | --- | --- | --- | --- | --- |
| Time (hr) | 12 | 24 | 48 | 72 | 96 |
| **P+P** | 1.24±0.03 | 0.87±0.05 | 0.91±0.06 | 0.62±0.02 | 0.71±0.02 |
| **+10% C** | 0.65±0.02 | 0.22±0.00 | 0.24±0.01 | 0.08±0.01 | 0.26±0.01 |
| **+1% C** | 1.41±0.06 | 0.65±0.00 | 0.39±0.06 | 0.48±0.00 | 0.72±0.00 |
| **+10% I** | 1.37±0.35 | 0.20±0.02 | 0.19±0.01 | 0.09±0.00 | 0.18±0.01 |
| **+1% I** | 1.23±0.13 | 0.39±0.00 | 0.60±0.04 | 0.50±0.01 | 0.81±0.06 |
| **+10% T** | 0.56±0.01 | 0.12±0.01 | 0.10±0.00 | 0.07±0.00 | 0.08±0.00 |
| **+1% T** | 1.46±0.25 | 0.37±0.04 | 0.95±0.07 | 0.55±0.02 | 0.83±0.02 |
|  |  |  |  |  |  |

**(B)**

| **IFNγ(ng/ml)** | | | | |  |
| --- | --- | --- | --- | --- | --- |
| Time (hr) | 12 | 24 | 48 | 72 | 96 |
| **P+P** | 0.01±0.13 | 0.16±0.11 | 0.62±0.06 | 4.56±1.12 | 8.07±0.90 |
| **+10% C** | 0.06±0.15 | 0.33±0.26 | 1.52±0.40 | 2.21±0.60 | 1.76±0.67 |
| **+1% C** | 0.01±0.19 | 0.19±0.05 | 3.04±0.07 | 4.21±1.24 | 3.73±0.52 |
| **+10% I** | 0.03±0.22 | 0.01±0.01 | 1.05±0.33 | 2.42±0.38 | 0.39±0.05 |
| **+1% I** | 0.09±0.21 | 0.10±0.00 | 1.62±0.09 | 5.19±0.41 | 2.11±0.09 |
| **+10% T** | 0.10±0.29 | 0.46±0.30 | 1.45±0.59 | 2.17±0.47 | 1.22±0.05 |
| **+1% T** | 0.16±0.11 | 0.32±0.14 | 2.45±0.61 | 2.72±1.32 | 5.65±0.44 |

|  |  | **IL-10(ng/ml)** | |  |  |
| --- | --- | --- | --- | --- | --- |
| **Time (hr)** | **12** | **24** | **48** | **72** | **96** |
| **P+P** | 0.24±0.02 | 0.78±0.13 | 1.55±0.05 | 0.88±0.03 | 0.99±0.05 |
| **+10% C** | 0.66±0.04 | 1.61±0.06 | 1.14±0.05 | 0.73±0.06 | 1.21±0.10 |
| **+1% C** | 0.23±0.04 | 1.23±0.07 | 1.23±0.05 | 1.02±0.02 | 0.69±0.06 |
| **+10% I** | 0.75±0.01 | 1.41±0.26 | 1.02±0.03 | 0.93±0.05 | 0.67±0.03 |
| **+1% I** | 0.33±0.03 | 1.04±0.05 | 1.15±0.02 | 0.75±0.03 | 0.76±0.01 |
| **+10% T** | 0.44±0.04 | 1.63±0.03 | 1.53±0.05 | 1.13±0.06 | 0.79±0.04 |
| **+1% T** | 0.21±0.02 | 1.03±0.00 | 1.17±0.07 | 0.77±0.03 | 0.66±0.06 |

|  | **IL-2(pg/ml)** | | | | |
| --- | --- | --- | --- | --- | --- |
| Time(hr) | 12 | 24 | 48 | 72 | 96 |
| **P+P** | 92.44±1.74 | 93.32±6.17 | 144.56±0.97 | 23.45±2.20 | 10.25±0.86 |
| **+10% C** | 62.11±1.83 | 107.02±9.44 | 134.63±2.49 | 17.23±1.27 | 10.36±0.28 |
| **+1% C** | 69.94±0.02 | 113.96±9.72 | 114.86±4.81 | 35.00±1.61 | 10.38±0.51 |
| **+10% I** | 89.80±6.04 | 119.44±4.66 | 116.31±5.99 | 13.36±0.23 | 9.19±0.81 |
| **+1% I** | 60.49±2.26 | 106.36±3.99 | 135.50±0.03 | 28.68±0.52 | 8.84±0.15 |
| **+10% T** | 126.55±1.02 | 190.88±11.15 | 206.36±13.05 | 86.25±6.56 | 8.64±0.01 |
| **+1% T** | 88.55±5.99 | 131.48±9.78 | 140.68±11.08 | 25.88±1.31 | 12.20±1.73 |

|  |  | **IL2-sR(ng/ml)** | |  |  |
| --- | --- | --- | --- | --- | --- |
| Time(hr) | 12 | 24 | 48 | 72 | 96 |
| **P+P** | ND | 0.09±0.00 | 0.81±0.10 | 3.08±0.23 | 5.51±0.27 |
| **+10% C** | ND | 0.35±0.04 | 4.52±0.02 | 3.27±0.57 | 5.19±0.32 |
| **+1% C** | ND | 0.21±0.04 | 0.98±0.24 | 3.27±0.41 | 5.21±0.29 |
| **+10% I** | ND | 0.17±0.09 | 2.55±0.46 | 3.08±0.98 | 6.05±0.40 |
| **+1% I** | ND | 0.17±0.06 | 1.53±0.34 | 3.48±0.81 | 5.18±0.59 |
| **+10% T** | ND | 0.27±0.02 | 1.07±0.11 | 5.12±0.59 | 5.77±0.07 |
| **+1% T** | ND | 0.22±0.08 | 0.95±0.07 | 2.98±0.25 | 5.96±0.32 |

|  |  | **TNF- α(ng/ml)** | |  |  |
| --- | --- | --- | --- | --- | --- |
| Time (hr) | 12 | 24 | 48 | 72 | 96 |
| **P+P** | 1.34±0.22 | 1.36±0.03 | 0.37±0.01 | 0.32±0.03 | 0.26±0.00 |
| **+10% C** | 0.47±0.02 | 0.21±0.00 | 0.05±0.01 | 0.02±0.00 | 0.04±0.00 |
| **+1% C** | 2.16±0.02 | 1.05±0.06 | 0.48±0.02 | 0.33±0.01 | 0.33±0.01 |
| **+10% I** | 0.51±0.02 | 0.15±0.00 | 0.08±0.06 | 0.04±0.00 | 0.03±0.00 |
| **+1% I** | 2.10±0.04 | 1.20±0.01 | 0.39±0.02 | 0.30±0.02 | 0.26±0.01 |
| **+10% T** | 1.42±0.01 | 0.57±0.02 | 0.22±0.01 | 0.10±0.00 | 0.07±0.01 |
| **+1% T** | 2.17±0.02 | 1.60±0.03 | 0.60±0.01 | 0.66±0.01 | 0.17±0.01 |

PBMCs were stimulated with PHA in the presence of different doses of either bone marrow (A) or Wharton’s jelly derived MSCs (B) which were either unprimed or primed with IFNγ (I) or TNFα (T) for different time intervals as indicated in the table. Cell supernatants were collected after defined time intervals and levels of secreted cytokines in different co-culture conditions were measured by specific BD OptiELISA kits. Each experiment was set up in triplicates. “+” indicates co-cultures of PHA stimulated PBMCs with appropriately treated MSCs as mentioned above. ND refers to not detected.
