# Supplementary figures and images for: Pro-Inflammatory Cytokines, IFNγ and TNFα, Influence Immune Properties of Human Bone Marrow and Wharton Jelly Mesenchymal Stem Cells Differentially
Source: PLoS One. 2010 Feb 2;5(2):e9016. doi: 10.1371/journal.pone.0009016 (PMC2814860; doi:10.1371/journal.pone.0009016)

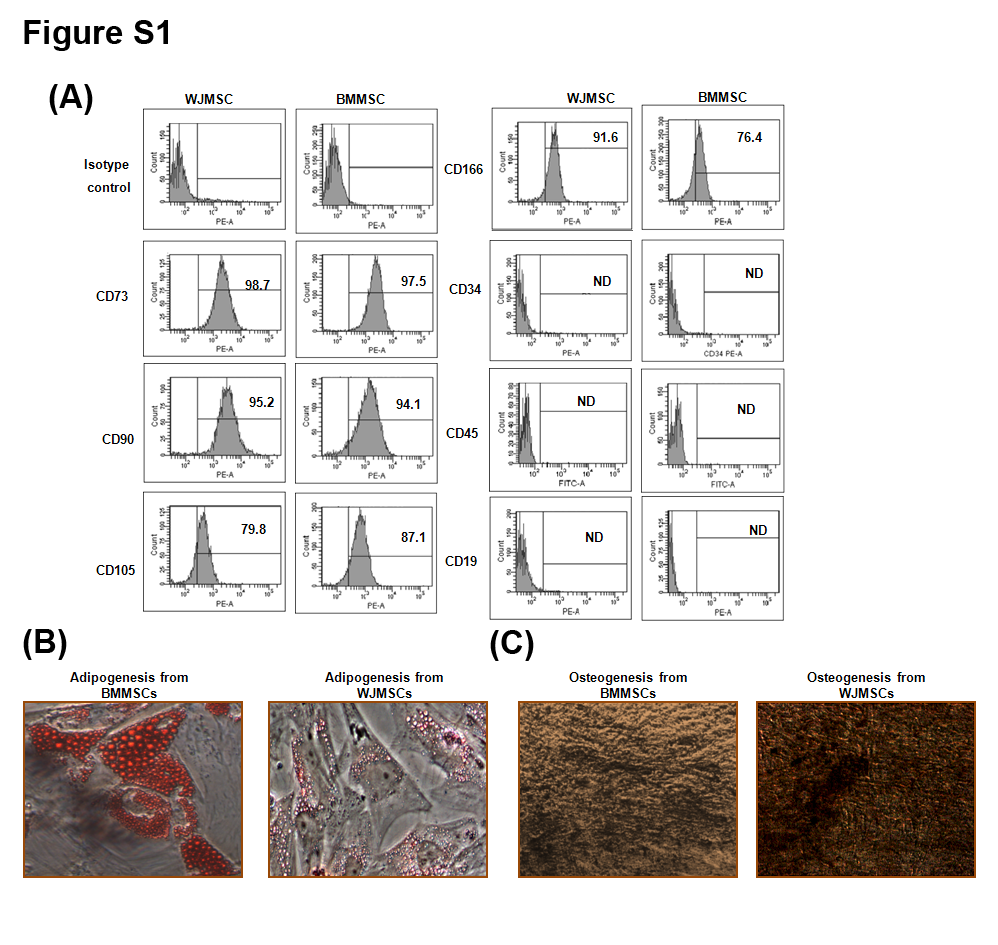

Supplement: Figure S1 — WJMSCs and BMMSCs exhibit conserved mesenchymal marker expression (A) and differentiation capacities (B, C). Mesenchymal marker expression at passage 5 is depicted. % positivity for each marker is shown in the figure and isotype control has been used for gating. ND-No positive population detected. (B) Represents Oil O Red staining of adipocytes induced from MSCs (magnification 10×40). (C) Represents Von Kossa staining of mineralized deposits after osteogenic induction of MSCs (magnification 10×10). (2.86 MB TIF) [file pone.0009016.s004.tif]

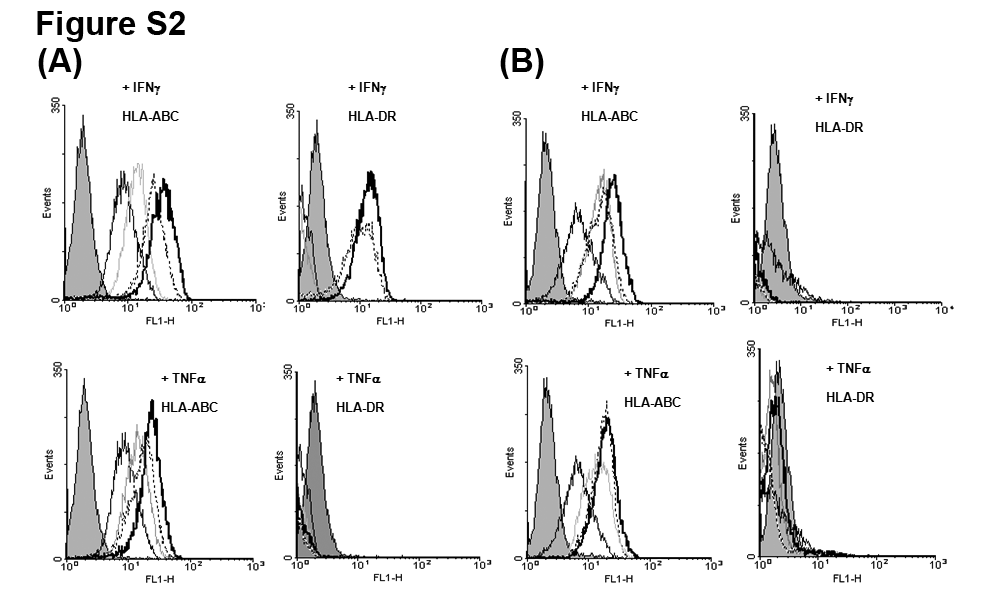

Supplement: Figure S2 — Kinetics of expression of HLA-ABC and HLA-DR on MSCs upon treatment with pro-inflammatory cytokines IFNγ and TNFα. BMMSCs (A) and WJMSCs (B) were treated or untreated (black line) with 150 U/ml of IFNγ or 10 ng/ml of TNFα for either 24 hr (grey lines), 48 hr (black dotted lines) or 72 hr (thick black line) and subsequently cell surface levels of HLA-ABC or HLA-DR was evaluated by Flow cytometry. Grey filled histogram represents staining with the matched isotype controls. (0.61 MB TIF) [file pone.0009016.s005.tif]

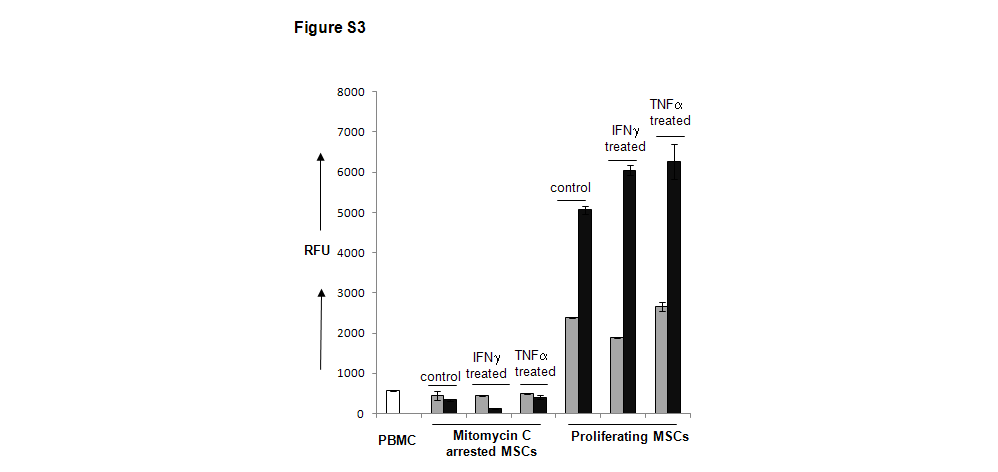

Supplement: Figure S3 — BrdU uptake of MSCs treated with Mitomycin C (10μg/ml) is comparable or lower than resting PBMCs in culture. The grey filled histogram represents BMMSCs whereas black filled histogram represents WJMSCs. RFU is relative fluorescence units after subtraction from endogenous fluorescence controls (-BrdU sample). (1.43 MB TIF) [file pone.0009016.s006.tif]

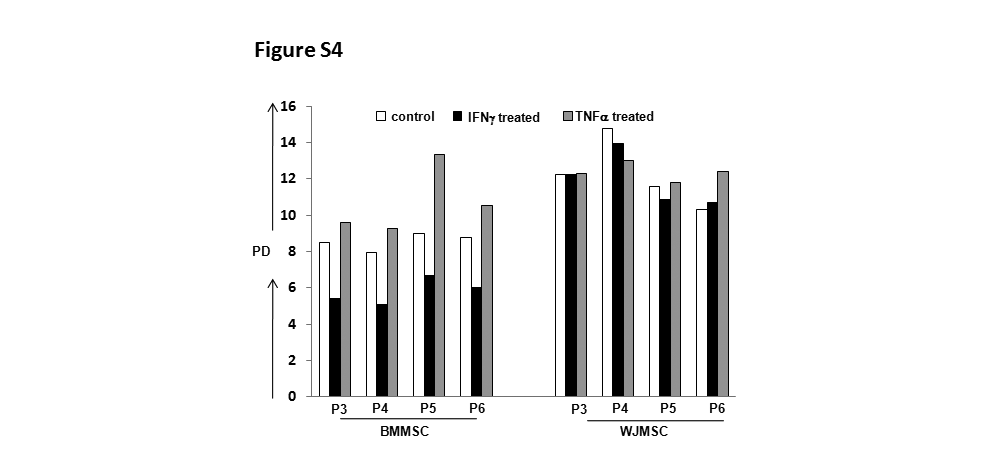

Supplement: Figure S4 — Exposure to IFNγ and TNFα alters the replication potential of BMMSCs. PD refers to population doublings calculated after priming with either IFNγ or TNFα at each passage (P) as indicated on the x-axis. (0.49 MB TIF) [file pone.0009016.s007.tif]

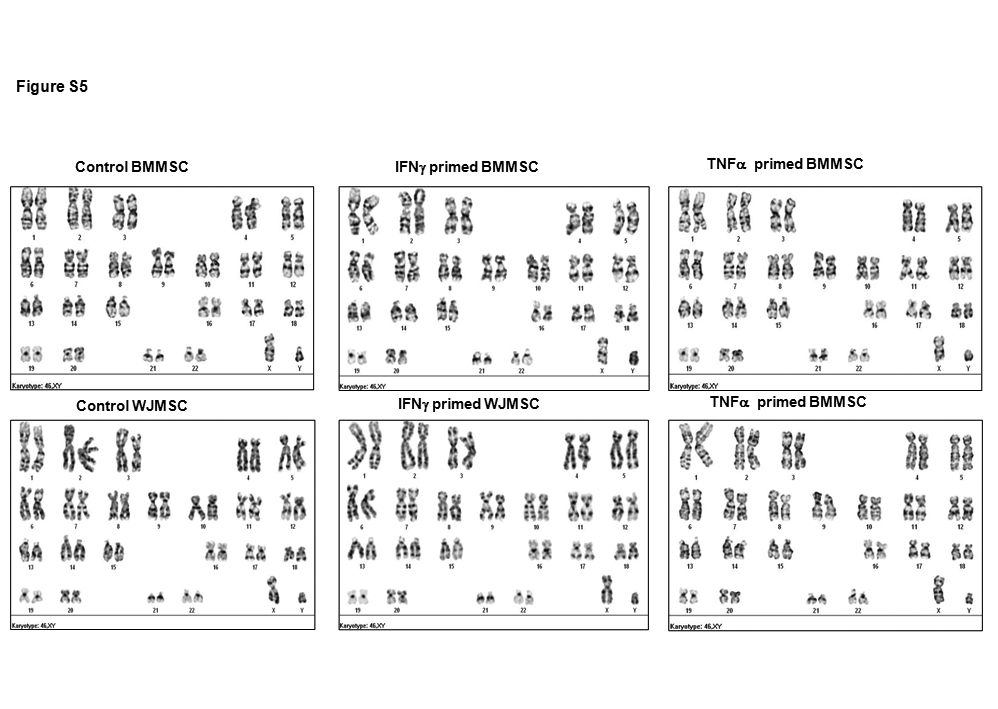

Supplement: Figure S5 — Normal karyotype of unprimed and primed WJMSCs and BMMSCs. Karyotype of Passage 5 MSCs is depicted. (0.73 MB TIF) [file pone.0009016.s008.tif]

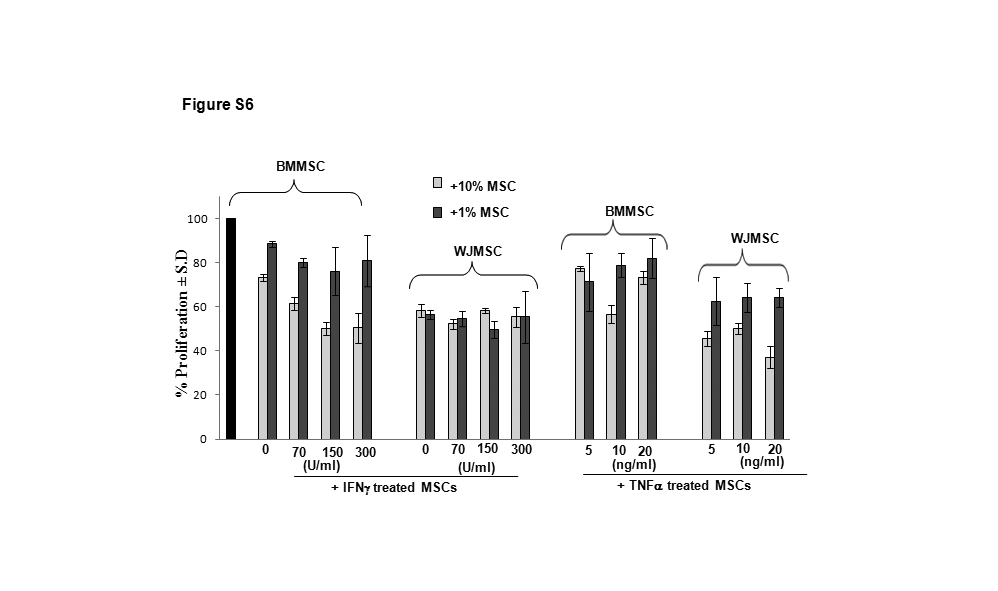

Supplement: Figure S6 — BMMSCs and WJMSCs retain their immuno-modulatory properties on exposure to varying doses of pro-inflammatory cytokines. C refers to proliferation in PHA treated PBMCs. “+” refers to co-cultures of PHA treated PBMCs and MSCs primed with different concentrations of IFNγ or TNFα. Representative experiment performed in triplicates is depicted above. (0.61 MB TIF) [file pone.0009016.s009.tif]
